# Enabling discovery of the social determinants of health: using a specialized lens to see beyond the surface

**DOI:** 10.5195/jmla.2025.2186

**Published:** 2025-07-01

**Authors:** Cynthia Sheffield, Gisela Butera, Dera Tompkins, Vence Bonham, Deborah Duran, Kimberly Middleton, Corina Galindo

**Affiliations:** 1 cynthia.sheffield@nih.gov, Biomedical Librarian, Office of Research Services, Division of Library Services, National Institutes of Health Library, Bethesda, Maryland, 20892 USA; 2 gisela.butera@nih.gov, Biomedical Librarian, Office of Research Services, Division of Library Services, National Institutes of Health Library, Bethesda, Maryland, 20892 USA; 3 dera.tompkins@nih.gov, Biomedical Librarian, Office of Research Services, Division of Library Services, National Institutes of Health Library, Bethesda, Maryland, 20892 USA; 4 bonhamv@nih.gov, Former Acting Director, National Human Genome Research Institute, Social and Behavioral Research Branch, Bethesda, Maryland, 20894 USA; 5 deborah.duran@nih.gov, Senior Advisor to the Director for Data Science, Data Analytics and Data Systems, National Institute of Minority Health and Health Disparities, Bethesda, Maryland, 20817 USA; 6 middletonk@cc.nih.gov, Former Principal Investigator, NIH Clinical Center, Translational Biobehavioral and Health Disparities Branch, Bethesda, Maryland, 20892 USA; 7 corina.galindo@nih.gov, Program Specialist, National Human Genome Research Institute, Bethesda, Maryland, 20892 USA

**Keywords:** Health care disparities, Social determinants of health, Controlled Vocabulary, Data Curation, crosswalk methodology, qualitative/quantitative analysis, evidence scan

## Abstract

**Background:**

Investigators encounter challenges in uncovering valuable studies when they are researching health disparities and minority health literature. This evidence scan and qualitative/quantitative crosswalk analysis looked at maternal health literature to gain a better understanding of the nuances in articulating the social determinates of health (SDoH) concepts aligned with the NIMHD Research Framework. SDoH concepts describe the multifaceted causes of health disparities, as opposed to effects that result in health outcomes.

**Methods:**

An evidence scan was conducted to identify literature for a health disparity population using infant low birth weight as a sample population. A qualitative and quantitative analysis of results was performed to examine the medical subject headings (MeSH) terms used to index the literature, along with the terminology used to describe various concepts related to the SDoH within the literature. A crosswalk of MeSH terms to SDoH concepts was used to see if a concentrated focus on SDoH concepts would improve discoverability of the literature.

**Results:**

The 31 articles selected demonstrated that 80% of the MeSH indexed keywords are unique within this collection of full text articles, despite the commonality of the topic. VOSviewer and a Python term counting program were used to visualize the diffusion of terminology. NVivo textual analysis revealed SDoH concepts within meaningful phrases within the literature. Major SDoH themes emerged from the analysis, although were not indexed. Authors used a crosswalk approach with SDoH concepts, to demonstrate that MeSH terms could be used to identify content with a more granular SDoH focus.

**Conclusion:**

Identifying literature that has SDoH concepts within the full text is difficult, due to the diffused nature of the terminology used to describe these concepts. This paper proposes to demonstrate how a crosswalk approach from MeSH terminology to SDoH concepts can provide a methodology for improving the discoverability of the literature. New technologies such as natural language processing, combined with existing technologies to normalize disparate ways of describing similar or related constructs, could be used to help discover and synthesize literature related to SDoH. Investigators, indexers, and librarians can work together to create an improved process for researchers.

## INTRODUCTION

Social Determinants of Health (SDoH) concepts describe the multifaceted causes of health disparities, as opposed to effects that result in health outcomes. Research related to health disparities and minority health is frequently sought by professionals who work to improve patient wellness, wellbeing, and good clinical outcomes for all. For decades investigators have tried to develop a consensus on how to describe health disparities concepts. Seminal work produced by the Heckler Report in 1984, focused on improving the poor health outcomes for minority populations, as well as the factors that contributed to these outcomes [1]. Experts have continued to call for standard terminology to describe social constructs, such as racism, that lead to health disparities [2-4].

There are many standards available to describe diseases and conditions, however, what is needed are recognized standards to capture SDoH language. In the 2021 publication, “The Science of Health Disparities Research,” experts noted a need for constructing consistent and standardized language, as well as collaboration among diverse research communities, and an interdisciplinary approach to studying health in minority and low socioeconomic groups [5]. This call to action was described by Duran and Pérez-Stable in 2019 to address the etiology of risk factors and how they impact health outcomes [6]. In their article, “Novel Approaches to Advance Minority Health and Health Disparities research,” the authors outline the causes of health disparities into five broad domains, with 12 to 27 SDoH concepts associated with each domain. The concepts describe the multifaceted causes of health disparities, which lead to health outcomes, such as “*low birth weight*” or “*preterm birth*.” This well-defined structure of health determinant concepts was developed by leading experts in the field. Further work culminated in producing the National Institute on Minority Health and Health Disparities (NIMHD) Research Framework [7].

In recent years, natural language processing (NLP) has been used to explore and identify SDoH terms from medical records. NLP analyzes textual data using computational methods to build a model of the text. It builds both syntactic and semantic models of text to provide structure to unstructured language. Syntactic models resolve ambiguities in the relationships between words. Information extraction looks for sematic associations within text, using an ontology to identify strings of text related to a specific topic [8].

A recent multicenter study noted the importance of uncovering this information to holistically assess clinical decision making needed during diagnosis and therapy planning [9]. The study used NLP techniques to identify a narrow scope of health factors from medical records. The authors of the paper developed a lexicon using manual curation, as there was not an established gold standard terminology fit for use. Keyword matching and classification was a primary method for identifying terminology [9].

In 2013, Bekhuis et al. studied the topic of “comparative effectiveness research design” (CER). Authors of the study worked with experts to develop a local terminology. Bekhuis et al. then aligned medical subject headings (MeSH) terms with their newly developed taxonomy of CER terms by crosswalking the local terminology, to MeSH and Emtree terms [10]. Crosswalking is the mapping of equivalent or near equivalent elements within a database schema. This methodology presented itself as a solution to resolve the frustration experienced by SDoH researchers.

In this study, we adapted the crosswalk approach to bring the SDoH concepts and MeSH terms together to improve discoverability. Previous studies have noted there is potentially more granularity within the Emtree taxonomy [11], however there were compelling reasons to focus on MeSH for this study. Improvements have been made in indexing medical terminology, as the National Library of Medicine (NLM) added SDoH terms to the MeSH taxonomy in 2020 [12]. In a recent November 2024 study, Suda-King et al. [12] described the improved precision and recall for high priority SDoH terms and definitions. The importance of their findings conveys one of several ways emerging studies are being indexed.

Authors of this paper began this research after a bibliometric portfolio analysis on SDoH topics was performed. The analysis returned limited SDoH terms. A broader investigation showed why societal factors that influence health may have been omitted and how the creation of relevant MeSH terms proved to be a larger undertaking. Authors posit the crosswalk of MeSH terms to the SDoH concepts will provide a tool for librarians and investigators. It will also demonstrate the need for further methods of discoverability, using emerging technologies to facilitate rapid and precise retrieval of SDoH concepts throughout the literature.

We conducted an evidence scan of maternal and child health studies and qualitative/quantitative analysis of the literature and performed a crosswalk of the results. To ensure the quality and intention of this evidence scan and crosswalk of terminology, leading experts, and practitioners in the field from the National Institutes of Health agreed to participate as committee members for the protocol. These committee members offered meaningful insights, which enabled a narrower scope and focus. Their recommendation to focus on a well-defined outcome, “*low birth weight*,” provided the precise scope, to enable ample SDoH terms to emerge from the literature. They also contributed and advised throughout the process. The aim of this evidence scan and quantitative and qualitative analysis is to explore how SDoH terminology is being used, and how it aligns with the NIMHD Research Framework [7], and how SDoH concepts could be better aligned with MeSH terminology.

## METHODS

To examine the SDoH terminology, we used three distinct methodologies: an evidence scan, quantitative and qualitative textual analysis, and a crosswalk to MeSH terms to SDoH concepts.

### Evidence Scan

We performed a review of the literature to answer the question “what the relationship is between health disparities and low birth weight?”, focusing on low birth weight within the broader category of maternal and child health literature.

The evidence scan methods which were adapted from the National Academies of Sciences, Engineering, and Medicine 2020 review process, uses an analytical framework to develop the associations between maternal/low birth weight and SDoH factors [13, 14].

Similar to a systematic review methodology, an evidence scan uses a rigorous approach to searching the literature, screening records to promote transparency and reproducibility, but it does not include an evaluation of the results or risk of bias. The analytical framework provides the inclusion and exclusion criteria and limits to the review. A protocol was developed and registered in Open Science Framework, https://osf.io/zw7a3.

The inclusion and exclusion framework was developed in consultation with content expert committee members. The topic of health disparities and low birth weight is well researched within the literature, and preliminary searches discovered existing quality systematic reviews. Therefore the authors decided to take an umbrella review approach to this evidence scan by limiting the search to systematic reviews, scoping reviews, umbrella reviews and meta-analysis. The search date was limited to 2019-2014 to capture current use of SDoH terminology and to include literature prior to the Covid pandemic. The authors wanted to see if there were any trends that could be noted pre-Covid pandemic versus post-pandemic. We also wanted to see if any of the SDoH MeSH terms added in 2020 were used or if more frequent indexing of SDoH terms appeared. The inclusion and exclusion framework shows the *a priori* criteria used to examine and determine the selected articles.

Inclusion criteria:

- *Population:* pregnancy (prenatal care, labor and delivery, difficult pregnancy care, eclampsia, ectopic, high-risk). premature. Birth—newborn (4 weeks, 28 days) (birth, newborn)
- *Intervention:* health inequity, structural racism, access to healthcare, social determinates of health
- *Comparison*: None
- *Outcome:* low birth weight. Preterm, low gestational weight/small fetus size
- *Study Design:* systematic reviews, meta-analysis, scoping, rapid, umbrella reviews. published peer-reviewed articles
- *Limits*: Human. English, United States
- *Date*: 2019–2024

Exclusion criteria:

- *Population:* children older than 28 days, paternal/father health outcomes, maternal studies not including pregnancy and newborn
- *Intervention:* studies not focused on health disparities or marginalized groups or studies not focused on access to healthcare
- Studies not focused on delivery of healthcare
- *Comparison*: None
- *Outcome:* assessment/measurement of scale, survey, or instrument, or focused on government policy without addressing health disparities
- *Study Design:* literature or narrative reviews, or non-review articles, non-peer-reviewed articles, (e.g., preprints, conference abstracts, proceedings, dissertations, reports, editorials, and retractions) or no abstract available
- *Limits*: animal studies, non-English, and studies that do not include US data
- *Date*: studies published before January 2019

The search strategy was developed by medical librarians (GB, DT, and CS) in collaboration with the committee members (VB, DD, CG, and KM). The comprehensive search strategy included keywords, MeSH and Emtree terms using Boolean operators and the strategy was peer reviewed. The search was conducted on February 22, 2024, in two electronic biomedical databases:

PubMed/MEDLINE and Embase (Elsevier). (See Appendix A: Search Strategies). The search strategy is in searchRxiv: https://www.cabidigitallibrary.org/doi/10.1079/searchRxiv.2025.00935.

Database results were exported into EndNote X21 (Clarivate) reference manager and after the removal of duplicates, records were imported into Covidence screening software (Covidence, Veritas Health Innovation, Melbourne, Australia; available at www.covidence.org). An initial pilot using sample records was performed by two reviewers (CS and GB) in Covidence to help refine the eligibility criteria and data extraction, and to ensure consistency applying the inclusion and exclusion criteria. The full screening of records was completed by two reviewers (CS and GB) who independently screened title/abstract and the full text of records, applying the inclusion criteria. Conflicts were resolved by a third reviewer (DT).

### Quantitative and Qualitative Analysis

Three methods of analysis were used to examine the relationship between terminology used to describe the SDoH and the SDoH as concepts. First, a keyword co-occurrence analysis of the literature results was conducted using VOSviewer (version 1.6.18, bibliometric network analysis software), to visualize the relationships between terms. VOSviewer is an open-source co-occurrence analysis tool that produces reliable and clear visualizations of bibliometric networks. In this process, the citations of included articles were identified within Clarivate Web of Science Core Collection, using PubMed ID numbers. The citations were then exported using the “full record and cited references” format and moved to VOSviewer, where a keyword co-occurrence algorithm was run. Keyword co-occurrence is the number of publications where keywords, extracted from the title, abstract, or author keyword list, appear in two or more publications [15]. Visualized maps were generated from this analysis, to show the frequency and relationships between terms.

The second method applied a “bag-of-words” approach, which is a technique used within NLP. The “bag-of-words” approach counts how often a particular term is used in a collection of documents; by figuratively tossing the terms into a “bag” and pulling each term out to count how frequently the term appears within the collection of documents. The process provided a representation of terminology used within the result set. MeSH terms were uploaded to a Python script to count the number of occurrences for each MeSH term across the data set. The code and associated data are available in Open Science Framework, https://osf.io/nge2s.

In the third method, a qualitative analysis was used to identify text describing the SDoH within the full text of included articles. NVivo 14 (Lumivero) allows researchers to structure code to identify and qualitatively assess themes within unstructured text, and was used for the analysis because the software was available to the authors. The full text of the 31 articles were imported into NVivo and two reviewers (CS, GB) independently performed qualitative analysis by mapping phrases that contain health determinants as contributing factors in adverse health disparity outcomes.[6] In this study these health determinants are referred to as SDoH concepts, and the domains for these health determinants or concepts align with the NIMHD Minority Health and Health Disparities Framework.[7] To help ensure coding consistency between reviewers, an initial pilot was performed using 20 sample articles, with a subset of SDoH concepts. The full set of SDoH concepts in each of the five domains within the NIHMD Framework were used to code the final included full-text articles. After the coding process was completed in NVivo, results were reviewed by both screeners (CS, GB) with a third reviewer (DT) present to settle disputed coding items. A coding comparison was performed.

### Crosswalk of MeSH Terms to SDoH Concepts

Authors of this paper wanted to know how many SDoH concepts aligned directly with one or more medical taxonomies. A crosswalk model used by Bekhuis, et. al [10] was adapted to map MeSH terms to the SDoH concepts as described by Duran and Pérez-Stable in 2019 [6]. Their description of domains and associated concepts provided the ideal structure for mapping MeSH to the NIMHD Research Framework since both knowledge structures were developed and maintained by NIH. The decision to focus on MeSH was based on the opportunity to explore the use of newly established SDoH MeSH terms within the literature[12], and NLM's easy to use *Mesh on Demand* tool. The NLM, *MeSH on Demand[16]* and *the MeSH Browser[16]* were used to confirm the definitions of terminology as they were applied to concepts. If a series of terms branched up into a higher-level term within the MeSH tree structure, then the higher-level term was listed.

A comparison of crosswalked SDoH terms and MeSH terms indexed to articles was conducted on a subset set of articles to see if there was a variance. The subset of articles were chosen based on their ability to represent the major themes extracted from the NVivo thematic analysis. SDoH concepts for each article were listed. The “crosswalked MeSH terms” were matched with SDoH concepts identified in the subset of articles. The crosswalked MeSH terms were then compared with MeSH terms indexed within PubMed to see if there was a variance in the two lists of terms.

## RESULTS

### Evidence Scan

**Figure 1:**
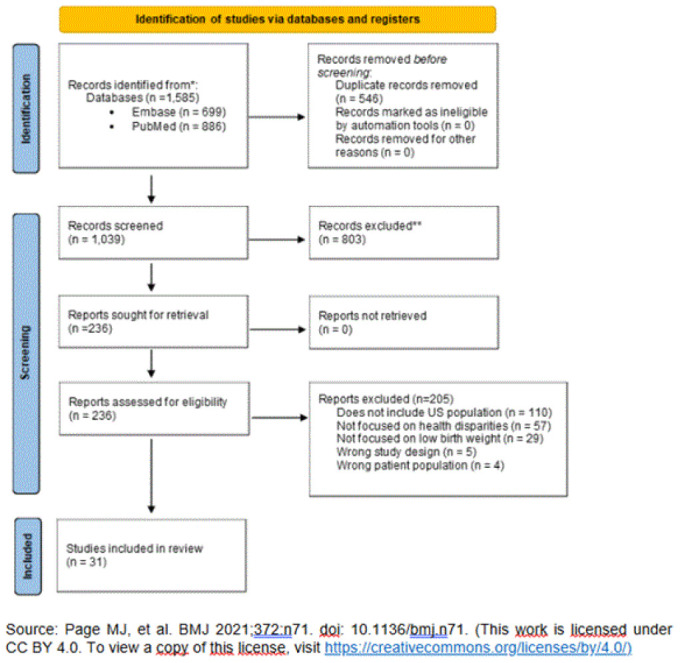
PRISMA flow diagram to show studies included in the review

The database search results generated 1,585 records and after the removal of duplicates, 1,039 abstracts were screened, with a total of 236 articles were included for full-text review, and a final 31 articles [17-47] meeting the inclusion criteria (Figure 1). Of the 31 studies identified four were scoping reviews, 14 were systematic reviews and 13 were systematic reviews and meta-analyses. The full list of included articles is in Appendix B: Table 1 - Study Characteristics.

### Qualitative Assessment of Keywords Used in the Literature

VOSviewer Keyword Co-occurrence Networks provide knowledge maps of how topics relate to each other in a particular field. Nodes are the keywords, and lines show the number of keyword co-occurrence connections within a collection of publications. The size of a node indicates the number of publications affiliated with that keyword, and the thickness of the lines shows the number of co-occurrences. The algorithm clusters keywords that are used frequently together with the same color and in proximity to how frequently the terms appear together.

The VOSviewer keyword co-occurrence analysis was performed using 29 of the 31 citations identified during the evidence scan. The algorithm for “All Keywords” was used, which contains both, “Author Keywords” and “Keywords Plus.” “Keywords Plus” includes words or phrases that appear in the titles of an article's references, but do not appear in the title of the article itself. Since these keywords are based on titles of articles in the reference list, articles without references will not contain Keywords Plus.[48] Two citations (Bellerose, 2022, DiTosta, 2021) did not produce “Keywords Plus” from the Web of Science, Core Collection, thus these citations were not included in the analysis.

A 2-keyword term analysis, where a keyword needs to occur at least twice in a set of publications, provides a good landscape map on a given topic. Appendix C, Figure 1, shows “*preterm birth*” is the most frequently used keyword, as expected, since it is central to the topic. A few SDoH concepts can be viewed on the perimeter of the various clusters within the landscape map. Climate related terms appear in the yellow cluster. The red cluster shows relationships between SDoH concepts such as “*racial/ethnic disparities*,” “*psychosocial factors*.” The green cluster lists “*ambient air-pollution*,” and “*intimate partner violence*.” From the collection of articles, 30 Keyword Plus terms appear within the landscape map. Nine of those terms are associated with risk factors that align with SDoH concepts.

If we readjust the algorithm to examine the landscape for more granularity at 3 Keyword Plus terms, where a Keyword Plus term has to occur in a set of publications a minimum of three times, we find the image generated (shown in Appendix C, Figure 2) produces a map with 15 or half of the Keyword Plus terms. The keyword term, “*preterm birth*” continues to appear central as expected, but the SDoH concept terms have almost disappeared. The only two terms that align with SDoH concepts are “*racial/ethnic disparities*” and “*high ambient-temperature*.”

### Quantitative Analysis of the Usage of MeSH Terms

MeSH terms from the 31 studies were also examined using a “bag-of-words” approach to measure the frequency of use for each term. The results from the “bag-of-words” analysis demonstrated how SDoH terminology is diffused, as over 80% of the terms were used only once or twice, within the 31 articles. The list of terms below in Table 1 shows the terms and how often each term appears within the 31 studies. There were 100 unique MeSH terms from the 31 included articles. Most MeSH terms were used fewer than 10 times. Most terms were used once (70 terms) or twice (11 terms).

**Table 1:** Number of times each MeSH term was used to index an article in the result set of 31 studies

27	Humans
23	Females
23	Pregnancy
21	Infant, Newborn
20	Premature Birth
11	Infant, Low Birth Weight
10	Pregnancy Complications
8	Infant
6	Stillbirth
5	Risk Factors Pregnancy Complications
4	Developed Countries Socioeconomic Factors
3	Prenatal Care Adult Cross-Sectional Studies Racism Birth Weight
2	Social Determinants of Health Observational Studies as Topic Outcome Assessment Health Care Hot Temperature Maternal Health Services Child Cesarean Section Mothers Parturition Infant, Small for Gestational Age Air Pollution
1	Pre-Eclampsia Pregnancy, Unplanned Poverty Areas Poverty Physical Exertion Perinatal Death Patient Protection and Affordable Care Act Pregnancy in Adolescence Particulate Matter Overweight Abortion, Spontaneous Prenatal Exposure Delayed Effects Systematic Reviews as Topic White People Walking Violence Veterans Temperature Stress Disorders, Post-Traumatic Prospective Studies Standing Position Sociodemographic Factors Social Work Social Support Social Environment Retrospective Studies Racial Groups Microaggression Occupational Exposure Developing Countries Health Services Accessibility Food Insecurity Follow-Up Studies Fetal Growth Retardation Extreme Heat Ethnic and Racial Minorities Employment Diabetes, Gestational Depression, Postpartum Hearing Tests Demography Cohort Studies Child, Preschool Child Nutritional Physiological Phenomena Black or African American Anxiety Disorders Air Pollution Hearing Hispanic or Latino Obesity Maternal Exposure Nutrition Assessment Neonatal Screening Military Personnel Military Deployment Adolescent Medically Uninsured Medicaid Maternal Health Maternal Age Housing Lifting/adverse effects Life Change Events Insurance, Health Insurance Coverage Infant, Premature Infant Nutritional Physiological Phenomena Infant Health Indigenous Peoples Women, Working

The authors were curious to know if any differences in indexing could be determined for those publications that emerged just prior and during the pandemic, versus those articles published as we were recovering from the pandemic. The 31 results were divided by years of publication into 2 sets to explore those differences: articles published from 2019–2021 and those published between 2022–2024. Authors conjectured that research published prior to 2021 was likely conducted prior to or early on in the Covid pandemic. Publications published during or after January 2022, had the opportunity to use the updated NLM SDoH terms.[12] Twelve articles from the result set were published between 2019–2021. Nineteen articles were published between 2022–2024. Two articles [28, 33] did not have MeSH terms applied within PubMed. A basic comparison of the number of SDoH Terms indexed was used as a rudimentary barometer to compare indexed MeSH terms: One to 2 terms; versus 3 or more terms. Of the 12 articles published between 2019 and 2021, 9 (75%) had 1 or 2 SDoH related MeSH terms. Three articles (25% of reviewed articles) contained between 5 and 7 terms. Of the 19 articles published between 2022–2024, 15 (79%) only had 1 to 2 SDoH related MeSH terms. The 4 other articles (21% of reviewed articles) had 3 to 7 terms listed. Based on this simple analysis no significant trends were noted in the use of MeSH terms.

### Qualitative and Quantitative Assessment of SDoH Terms

The NVivo analysis reveals that SDoH themes within the full text of the 31 selected articles retrieved by the evidence scan matched the 5 domains of influence from the NIMHD Framework (*Biological, Behavioral, Physical/Build Environment, Sociocultural Environment, Health System Care*). This coding structure allowed us to see the volume of meaningful phrases in the selected articles that match the concepts within each domain. (See Appendix D, Figures 1 – 5)

Four dominate themes emerged from the studies: “*In utero exposure*,” *“Social and economic adversity*,” “*Discrimination, racism, and stigma*,” and “*Access to preventive service and quality health care*.” Examples of these four themes are in Appendix E, Table 1-Primary Coded Concepts. Additional phrases that relate to the various SDoH concepts can be viewed in the Appendix E, Table 2 – SDoH Coded Concepts, where they are organized by SDoH domains.

### Comparing Crosswalked MeSH Terms to SDoH Concepts

The crosswalk of MeSH Terms to SDoH concepts includes the five domains of Biological, Behavioral, Physical/Build Environment, Sociocultural Environment, Health System Care (See Table 2a-2e) and it demonstrates that most SDoH concepts have one or more MeSH terms that align with each concept. There are five SDoH concepts that did not map directly to MeSH terms. (See Table 3).

**Table 2a:** Behavioral, Crosswalk of MeSH Terms to SDoH Concepts

Behavioral	MeSH
Diet and nutrition	Physiological Phenomena Diet, Food, and Nutrition Beverages Fermented Foods Food Nutritional Physiological Phenomena
Preventive health behaviors	Health Behavior Health Risk Behaviors Self-Examination Breast Self-Examination Preventive Health Services Sleep Hygiene Smoking Cessation Smoking Reduction Tobacco Use Cessation Treatment Adherence and Compliance
Unprotected sexual intercourse	Unsafe Sex
Domestic/family violence	Domestic Violence Violence Exposure to Violence
Physical activity	Exercise
Substance use, abuse, misuse, and addiction	Substance-Related Disorders
Compliance and adherence with prescribed therapy	Treatment Adherence and Compliance
Delays in seeking care after symptom awareness	Refusal to Participate Attitude to Health Treatment Adherence and Compliance Patient Participation Treatment Refusal
Living responsibly with infectious disease	**No direct alignment with MeSH**
Hygiene/oral hygiene	Oral Hygiene Oral Health Dental Care Hygiene
Cultural beliefs	Culture Ethnic and Racial Minorities Family Hierarchy, Social Medicalization Minority Groups Secularism Social Capital Social Change Social Conditions Social Environment Social Class Social Factors
Religious beliefs/schemas	Religious Philosophies

**Table 2b:** Biological, Crosswalk of MeSH Terms to SDoH Concepts

Biological	MeSH
Biochemical	Biochemistry Biochemical Phenomena Biological factors Molecular Biology
Genome and epigenome	Genome
Proteome	Proteome
Microbiome	Microbiota
In utero exposure	Embryonic and Fetal Development
Metabolic factors	Metabolism
Pathogens	Disease Transmission, Infectious
Physiologic responses to stress/allostatic load	Allostasis
Organ systems	Cardiovascular System Lymphatic System Respiratory System Endocrine System Gastrointestinal Tract Urogenital System Musculoskeletal System Nervous System Immune System
Nervous system	Nervous System
Telomere/cellular aging/senescence	Telomere Cellular Senescence
Cellular functions and communication	Cell Physiological Phenomena
Enzymes	Enzyme Activation Enzymes
Inflammation	Inflammation
Demographics	Demography Quality of Life, Ethnicity
Endocrine system/hormones	Endocrine System Hormones

**Table 2C:** Clinical Events & Health Care System, Crosswalk of MeSH Terms to SDoH Concepts

Clinical Events & Health Care System	MeSH
Patient-clinical communication	Health Communication
Health insurance coverage/policies	Insurance, Health
Access to prevent services and quality health care	Health Services Accessibility
Disease management and functioning status	*Disease Management
Symptom and pain management	Palliative Care Pain Management
Drug interactions and synergies	Drug Interactions
Use of alternative therapies	Complementary Therapies
Appropriate diagnostics	*Diagnosis
Access to emerging techniques	Biomedical Technology
Access to public health education, information, and health alerts	Health Education
Precision medicine	Precision Medicine
Generalizability of research findings	Outcome Assessment, Heath Care
Translation of research	Culturally Appropriate Technology Disruptive Technology Technology Transfer
Dissemination and diffusion of research results	Diffusion of Innovation
Macro-structural stressors (e.g. Policies and procedures)	Social Control Policies
Incorporation of spiritual and traditional healers	Traditional Medicine Practitioners
Institutional discrimination in health care	Systemic racism Health inequities Minority Health Social Determinants of Health
Health care system mistrust	Betrayal Delivery of Health Care
Culturally competent care	Cultural Competency
Workforce diversity	Workforce diversity
Electronic medical records	Electronic Health Records
Palliative and end-of-life care	Palliative Care Terminal Care Hospice and Palliative Care Nursing
Living with chronic illness and comorbid conditions	Chronic Disease
Long-term care	Long-Term Care
Access to health information/consent in primary language	Consumer Health Information Patient Education as Topic
Policies and political practices	Health Policy
Diversity of biomedical/health delivery workforce	Health Workforce Workforce Diversity Diversity, Equity, Inclusion

**Table 2d:** Sociocultural Environment, Crosswalk of MeSH Terms to SDoH Concepts

Sociocultural Environment	MeSH
Employment status and security	Employment
Income	Income
Housing and food security	Housing Instability Food Supply Access to Healthy Foods Foods, Specialized
Health insurance status (affordability/quality)	Insurance, Health Medically Uninsured
Social and economic adversity and inequality	Socioeconomic Factors Economic Factors
Immigration and legal status	Transients and Migrants Human Migration
Geographic location	Geography Geography, Medical Topography, Medical
Residential segregation	Residential Segregation
Education attainment	Educational Status
Access to quality education	**No direct alignment with MeSH**
Transportation options	**No direct alignment with MeSH**
Limited English proficiency	Communication Barriers
Health literacy/numeracy	Health Literacy
Discrimination, racism, and stigma	Social Discrimination
Health socialization and education	Health education
Psychosocial stressors	Stress, Psychological
Historical trauma	Historical trauma
Social safety net	Safety-net Providers
Community reentry (e.g. prison, military service)	**No direct alignment with MeSH**

**Table 2e:** Physical Environment, Crosswalk of MeSH Terms to SDoH Concepts

Physical Environment	MeSH
Housing status	Housing
Neighborhood violence	No direct alignment with MeSH
Unhealthy housing units	Housing Residence Characteristics Home Environment
Residence crowding	Housing Housing Quality
Exposure to toxic substances (e.g., pollution, radiation, lead, mold, dust mites)	Environmental Pollutants Pesticides Radiation injuries Heavy Metal Poisoning Fungi Pyroglyphidae
Aesthetic elements (e.g. trees)	Environment Design Ecosystem
Access to safe recreational facilities	Sports and Recreational Facilities
Quality of air and water	Air Pollution Water Pollution
Concentration of fast-food outlets and access to full-service grocery stores	Fast Foods Food Supply Supermarkets
Public safety (e.g., fire dept., police)	Emergency Responders
Occupational conditions and hazards	Occupational Exposure Environmental Monitoring Radiation Exposure
Affordability of resources	Resource Allocation Economics

**Table 3:** SDoH Concepts that do not have corresponding MeSH terms

SDoH Domain	Concept
Behavioral	Living responsibly with infectious disease
Physical Environment	Neighborhood violence
Sociocultural Environment	Access to quality education
Sociocultural Environment	Community reentry (e.g. prison, military service)
Sociocultural Environment	Transportation options

### Comparing Crosswalked MeSH Terms to Actual MeSH Terms

A subset of six papers of the 31 studies was used for additional analysis. The subset articles were chosen based on their ability to represent the prominent themes that emerged from the textual analysis. The “crosswalked MeSH terms” were matched with the SDoH concepts identified in the textual analysis. The crosswalked MeSH terms were then compared with MeSH terms indexed in PubMed. The SDoH concepts, the crosswalked MeSH, and the actual MeSH are presented for these six articles in Table 4. These concepts are shown in groups representative of the five SDoH domains: Behavioral, Sociocultural Environment, Biological, Physical Environment, Clinical Events and Health Care System.

**Table 4:** Comparing Crosswalked MeSH Terms to Actual MeSH

Study	SDoH domains	SDoH Concepts Extracted from Qualitative Analysis	Crosswalked MeSH to SDoH Concepts	MeSH Terms Indexed in PubMed	Mesh Terms indexed in PubMed (non-SDoH)
Bellerose, 2022 [22]	Sociocultural Environment	Health insurance status (affordability quality) Social and economic adversity and inequality Health socialization and education Health literacy numeracy	**Insurance, Health ** **Socioeconomic Factors ** **Economic Factors ** **Health Education ** **Health Literacy**	**Insurance, Health ** Medicaid* Patient Protection and Affordable Care Act*	Adult Female Humans Infant, Newborn Pregnancy United States
	Physical Environment	Affordability of resources	**Resource Allocation**	**Health Services Accessibility**	
	Clinical Events and Health Care System	Health insurance coverage policies Long-term care Policies and political practices Health care system mistrust Institutional discrimination Access to preventive services and quality health care	**Long-term Care ** **Health Policy ** **Delivery of Health Care ** **Betrayal ** **Systemic Racism ** **Health Services Accessibility**	**Medically Uninsured ** **Health Services Accessibility ** **Insurance ** **Coverage**	
Crawford, 2021 [22]	Behavioral	Cultural beliefs/schemas	**Culture ** **Ethnic and Racial Minorities ** **Family**		Anxiety Disorders Female Humans Infant, Newborn Outcome Assessment, Health Care Pregnancy Premature Birth* United States
	Sociocultural Environment	Discrimination, racism, and stigma Historical trauma Health insurance status (affordability/quality) Income Social safety net Immigration and legal status Housing and food security	**Social Discrimination ** **Racism ** **Historical trauma ** **Insurance, Health ** **Income ** **Safety-net Providers ** **Transients and Migrants ** **Human Migration ** **Housing Instability ** **Access to Healthy Foods**	**Microaggression ** **Racism**	
	Biological	Physiologic responses to stress/allostatic load Metabolic Factors Telomere/cellular aging/senescence	**Allostasis ** **Metabolism ** **Telomere**		
	Clinical Events and Health Care System	Access to preventive services and quality health care Institutional discrimination in health care Policies and political practices Health care system mistrust Culturally competent care Diversity of biomedical/health delivery workforce	**Health Service Accessibility ** **Systemic Racism ** **Health Inequities ** **Minority Health ** **Social Determinants of Health ** **Medically Uninsured ** **Delivery of Health Care ** **Cultural Competency ** **Workforce Diversity**		
DiTosto, 2021 [30]	Sociocultural Environment	Housing and food insecurity Health insurance status Social and economic adversity and inequity	**Housing Instability ** **Food Supply ** **Insurance, Health ** **Socioeconomic Factors ** **Economic Factors**	**Housing**	Cross-Sectional Studies Female Humans Infant, Newborn Pregnancy Premature Birth Prospective Studies Retrospective Studies United States
	Physical Environment	Housing status Affordability of resources Residence crowding Unhealthy housing units	**Housing ** **Resource Allocation ** **Economics ** **Residence Characteristics**		
	Clinical Events and Health Care System	Access to preventive services and quality health care	**Health Services Accessibility**	**Outcome Assessment, Health Care**	
Dzekem, 2024 [31]	Behavioral	Tobacco use	**Tobacco Use ** **Tobacco Smoking**		Female Humans Infant, Low Birth Weight Infant, Newborn Pregnancy Pregnancy Outcome Premature Birth Stillbirth United States
	Sociocultural Environment	Education attainment Geographic location Social and economic adversity and inequality Discrimination, racism, and stigma Housing and food security Health insurance Social and economic adversity and inequality Income Social safety net Access to quality education	**Education Status ** **Geography ** **Socioeconomic Factors ** **Social Discrimination ** **Residential Segregation ** **Housing Instability ** **Food Supply ** **Insurance, Health ** **Socioeconomic Factors ** **Income ** **Safety-net Providers ** **Education**		
	Biological	Demographics Metabolic factors Biochemical Pathogens Cellular functions and communication	**Demography ** **Ethnicity ** **Metabolism ** **Biochemistry ** **Disease Transmission, Infectious ** **Cell Physiological Phenomena**		
	Physical Environment	Quality of air and water Exposure to toxic substances Residential segregation	**Air pollution ** **Water pollution ** **Environmental Pollutants ** **Quality of Life**	**Air pollution**	
	Clinical Events and Health Care System	Policies and political practices Generalizability of research findings Access to preventive health services and quality health care	**Health Policy ** **Outcome Assessment, Health care ** **Health Services Accessibility**		
Manzo, 2024 [36]	Behavioral	Living responsibly with infectious disease	**Communicable Diseases ** **Sexually Transmitted Diseases**		Female Humans Military Deployment Military Personnel Pregnancy Pregnancy Complications Pregnancy Outcome Premature Birth United States Veterans
	Sociocultural Environment	Psychosocial stressors Social and economic adversity and inequity Social safety net Community reentry Discrimination, racism, and stigma Health socialization and education Historical trauma	**Stress, Psychological ** **Socioeconomic Factors ** **Safety-net Providers ** **Reentry (non-MeSH) ** **Returning Citizen (non-MeSH ** **Social Discrimination ** **Health Education ** **Historical Trauma ** **Systemic Racism ** **Health Inequities**		
	Biological	Physiologic responses to stress, allostatic load	**Allostasis**		
	Physical Environment	Occupational conditions and hazards Neighborhood violence	**Occupational Exposure**	**Stress Disorders, Post-Traumatic**	
	Clinical Events and Health Care System	Long-term care Living with chronic illness and comorbid conditions Symptom and pain management Patient-clinical communication Access to preventative services and quality care Disease management and functioning status Institutional discrimination in health care Culturally competent care	**Long-Term Care ** **Chronic Disease ** **Pain Management ** **Health Communication ** **Health Services Accessibility ** **Disease Management ** **Health Inequities ** **Cultural Competency**		
van Daalen, 2022 [47]	Behavioral	Diet and nutrition Delays in seeking care after symptom awareness Tobacco use	**Diet, Food, and Nutrition ** **Attitude to Health ** **Treatment Adherence and Compliance ** **Tobacco Use**		Cohort Studies Cross-Sectional Studies Female Humans Infant, Newborn Pregnancy Pregnancy Outcome Premature Birth
	Sociocultural Environment	Discrimination, racism, and stigma Social and economic adversity and inequality Income Educational attainment Housing and food security Historical trauma Geographic location Psychosocial stressors Health socialization and education	**Social Discrimination ** **Socioeconomic Factors ** **Health Inequities ** **Income ** **Education Status ** **Housing Instability ** **Food Supply ** **Historical Trauma ** **Geography ** **Stress, Psychological ** **Health Education ** **Insurance, Health**	**Racism**	
	Biological	Demographics	**Demography**		
	Physical Environment	Affordability of resources Public safety	**Resource Allocation ** **Economics ** **Emergency Responders**		
	Clinical Events and Health Care System	Institutional discrimination in health care Institutional discrimination in health care Access to preventive services and quality of care Policies and political practices Institutional discrimination in health care Health insurance coverage policies Patient-clinician communication Macro-structural stressors (policies, procedures) Culturally competent care Workforce diversity Diversity of biomedical health delivery workforce Long-term care	**Health Services Accessibility ** **Health Policy ** **Systemic Racism ** **Health Communication ** **Social Control Policies ** **Cultural Competency ** **Workforce Diversity ** **Diversity, Equity, Inclusion ** **Long-Term Care**		

## DISCUSSION

The aim of this study was to explore how SDoH terminology is being used, how it aligns with the NIMHD Research Framework [7], and how SDoH concepts could be better aligned with MeSH terminology. Our data shows that, while the terminology used by authors aligns with the NIMHD Research Framework, authors are not using consistent terminology when writing about SDoH. We also learned that there are MeSH terms that align with SDoH terminology, but that they are not being applied to articles containing SDoH concepts. When we compared the Crosswalked MeSH to the actual MeSH (Table 4), we discovered a significantly more granular and specific level of SDoH that exist within MeSH.

The comparison of crosswalked MeSH to actual MeSH (see Table 4) demonstrates the differences between what SDoH concepts can be uncovered within the literature versus what concepts are being indexed. When using a SDoH focused approach several more MeSH terms can be applied to each citation. For instance the Bellerose [22] article has one of the highest rates of SDoH terms indexed within PubMed among the 31 articles included in this study. However, even with focused SDoH indexing in place, we identified several additional relevant MeSH terms such as: “*Economic Factors*,” “*Resource Allocation*,” “*Long Term Care*,” and “*Systemic Racism, Health Inequities*.”

This has significant implications for librarians and researchers who need to search the literature for SDoH concepts. The VOSviewer images in Appendix C showing the co-occurrence of 2 keyword terms (Appendix C, Figure 1) versus 3 keyword terms (Appendix C, Figure 2), begin to highlight the challenges librarians face as they develop search strategies that include SDoH concepts. The task is to cast a wide net, that includes a myriad of phases to capture a SDoH concept, while simultaneously maintain the specificity and precision to reduce the false hits within results, thus uncovering critical literature that contributes to health policy research.

The areas of SDoH terminology that are not covered by MeSH terms have a significant social science component to each concept, so it is understandable that they may not be found within the MeSH taxonomy. Although there are existing MeSH terms similar to these concepts, the terms are not directly correlated. For instance, “*neighborhood violence”* requires the combined use of MeSH terms: “*Residence characteristics*” and “*Violence*.” Whereas “*neighborhood violence”* refers to the level of distress within a community due to historic and current threat of violence.

Findings of this paper are consistent with similar studies that discuss the challenges of identifying SDoH terminology within the literature. Suda-King et al. noted the need for improved behavioral science research terminology to build a knowledge base[12]. We also observed that there are behavioral science terms that do not align well with MeSH terms and agree that there is a need for more clarity and definition around this terminology (See Table 3.).

Our results also confirm as Eldredge and Wallerstein did [49] that a deeper level of indexing for SDoH concepts can be applied to studies if researchers, librarians and indexers allow the SDoH concepts to emerge and be recognized within the paper, where they align with SDoH concepts, instead of assigning specific terms to a study.

This study shows why it is difficult to design a search strategy that will successfully uncover literature that describes SDoH concepts. The crosswalking exercise demonstrates that there are many existing MeSH terms available that align with SDoH concepts, yet, more importantly, how many SDoH concepts are being missed not just from indexing, but from discovery within the literature. A better process is needed to align existing MeSH terms with SDoH concepts or librarians and researchers will continue to miss relevant articles on SDoH.

The results from the VOSviewer analysis confirm that investigators are not describing SDoH concepts consistently. However, our qualitative analysis of the full text provides evidence these concepts are within the literature and that they can be mapped to the NIMHD Framework. Using the SDoH concepts as presented by Duran and Perez-Stable[6] can serve as a starting point to identify specific concepts for consideration, as part of an improved process. Librarians could use the SDoH concepts as a guide to generate “search hedges” for each concept, and associate the search hedges with the MeSH terms that were crosswalked to each SDoH concept. The SDoH concepts and corresponding MeSH terms could also be used by researchers to identify author keywords when publishing their work.

Many SDoH concepts are nuanced and the proper key terms could vary depending on the cultural or regional environment. A key term chosen to describe something in one environment, might be different than a term used within a similar but slightly different environment. For example, the circumstances to choose: “social discrimination,” “racism,” “microaggression,” or perhaps “historical trauma” may be similar but nuanced. We see the challenge not as defining and assigning the right terminology to the literature; rather the challenge is identifying SDoH concepts in their natural form within text and phrases, and then mapping them to identified MeSH key terms or other similar taxonomies. This process was completed in our study through a time-consuming and careful effort looking specifically for SDoH concepts. If health equity research is to continue to mature, this process needs to occur in a more automated fashion, where researchers can recognize SDoH concepts and associated terms in a less time intensive manner. There are decades of health science literature that contain a multitude of SDoH concepts, which provide evidence of health determinate risk factors. The research community cannot go back and reindex all of the literature, but we can create tools to enable the discovery of SDoH concepts within the literature, and link those concepts to existing taxonomies such as MeSH. This process will help researchers to identify health risk factors, all across the public health spectrum. Awareness that this alignment needs to occur is a step toward progress.

## FUTURE DIRECTIONS

In addition to librarians and researchers using the results of our research to identify concepts within the literature, indexers can now use our results to assign associated MeSH terms to specific studies, to improve the network of concepts across studies. Since this process is labor intensive and tedious, informaticians can work together with librarians, indexers, and investigators to explore how to leverage technology to improve discoverability of SDoH concepts. Recent improvements in MeSH indexing were reported by the Suda-King et al. study. They demonstrated significant improvements in precision within the quantitative and qualitative results. This precision is due to the result of adding 35 new SDoH terms to MeSH [12]. This is a good start, but our rudimentary analysis showed the newly added terms are not significantly effective on every topic. This finding reinforces the need for new tools, using techniques such as NLP to identify and tag SDoH concepts as they emerge from the literature, to then map those concepts to corresponding crosswalked terminology.

NLP used to uncover SDoH within medical records now[9] can be combined with other technologies at NLM to create such a tool. The terms, concepts, and sample phrases used to describe SDoH concepts identified in our analysis, can be used to create programmatic algorithms to identify concepts within text. To maximize the functionality of such a tool, a push and pull approach can be used to programmatically assess text. Search strings developed by librarians and researchers to identify SDoH concepts can be employed to push the search. The NLP syntactic and semantic models used to provide structure to unstructured language[8], can enable SDoH concepts to emerge, or pull, from the text. Such a dual action methodology could enhance technologies already used in other NLM tools.

The NLM provides a tool called PubTator 3.0 that was created to synthesize terminologies with similar content or context. It uses artificial intelligence to highlight semantic terms and their relationships within papers, allowing reviewers to filter and highlight entities such as diseases, chemicals, genes, or variants within these documents. PubTator 3.0 also uses autocomplete technology to normalize the nomenclature for a specific entity by pooling the various forms of chemical names or genes into a single format [50]. This study demonstrates that a similar tool, programmed to identify NLP text focused on each SDoH concept identified within the NIMHD Research Framework, would operationalize SDoH concepts [6]. It would be a valued resource used by researchers, librarians, and indexers to help normalize and solidify the terminology related to the SDoH.

This study has several limitations. We only examined MeSH terms in our analysis. Future research could include investigation of this crosswalk approach with Emtree terminology to examine the discovery of SDoH literature within Embase. We only applied the NIMHD Research Framework to analyze the SDoH literature and concepts. Exploration of SDoH concepts from other frameworks, such as the Center for Disease Control's Healthy People 2030 could be valuable. Our analysis has a narrow focus on maternal child health, in vulnerable populations, and only looks at “*low birth weight*”, “*small for gestational age*” or “*preterm birth*” as an outcome of pregnancy. Other topics may generate other SDoH concepts and varying numbers of results. Studies included populations either inside the United States, or international studies that included US populations which may limit generalizability. We did not include primary studies within the evidence scan, due to the overabundance of results of existing quality reviews. We limited the scan to systematic reviews, scoping reviews, umbrella reviews, rapid reviews, and meta-analysis. Primary studies may provide additional insight into how SDoH concepts are used within the literature. The synthesized reviews may characterize the SDoH concepts somewhat differently than reported in primary studies.

The results of our study showed MeSH terminology aligned with SDoH concepts provide a more granular lens for finding literature with a SDoH focus, but that current indexing does not reach its full potential. There is potential for this process to be automated and turned into an investigative tool to reveal embedded SDoH concepts across the health sciences literature by applying technology with leading experts in the field as well as bioinformatics experts at the NLM. This new tool would highlight SDoH concepts in the context of an article, and link them to associated MeSH terms. This would enable librarians and researchers to more readily identify, analyze and document SDoH health factors in context. It would also help indexers apply descriptive MeSH terms. This specialized lens could help health science investigators and informaticians demonstrate outcomes from the risks associated with health inequities, leading to effective preventive measures.

## Data Availability

Data and materials are available within the Supplementary information. The protocol will be made available at the Open Science Framework, https://osf.io/zw7a3.

## CODE AVAILABILITY

Code available at the Open Science Framework, https://osf.io/nge2s.

## SUPPLEMENTAL FILES

**Appendix A**: Search Strategies

**Appendix B**: Study Characteristics

**Appendix C**: Qualitative Assessment of Keywords Used

**Appendix D**: Qualitative Code SDoH Concepts

**Appendix E**: Tables 6a – 6b SDoH Concepts Emerged from Coding
