# Supplementary material for: Enabling discovery of the social determinants of health: using a specialized lens to see beyond the surface
Source: J Med Libr Assoc. 2025 Aug 1;113(3):204–22. doi: 10.5195/jmla.2025.2186 (PMC12369968; doi:10.5195/jmla.2025.2186)
Supplement: Supplementary file 1 — Appendix A: Search Strategies [file jmla-113-3-204-s01.docx]

# **Appendix A: Search Strategies**

Database: PubMed/MEDLINE

Platform: National Library of Medicine 
Date Searched: 2/22/2024

Date Limits: 2019-2024

|  | Concept: | Search Strategy: |
| --- | --- | --- |
| #1 | Birth | "Infant"[Mesh] OR "infan*"[Title/Abstract] OR "birth*"[Title/Abstract] OR "baby"[Title/Abstract] OR "babies"[Title/Abstract] OR "born"[Title/Abstract] OR "newborn*"[Title/Abstract] OR "new born*"[Title/Abstract] OR "newly born*"[Title/Abstract]  OR "Obstetric Labor, Premature"[Mesh] OR "prematur*"[Title/Abstract] OR "pre-matur*"[Title/Abstract] OR "preterm*"[Title/Abstract] OR "neonat*"[Title/Abstract] OR "neo-nat*"[Title/Abstract] OR "NICU"[Title/Abstract] OR "NICUs"[Title/Abstract] OR "premie*"[Title/Abstract] OR "preemie*"[Title/Abstract] OR "childbirth*"[Title/Abstract] OR "Delivery, Obstetric"[Mesh] OR "cesarean*"[Title/Abstract] OR "c-section*"[Title/Abstract] OR "induced labor*"[Title/Abstract] OR "vaginal delivery"[Title/Abstract:~4] OR "labor delivery"[Title/Abstract:~4] OR "vaginal deliveries"[Title/Abstract:~4] OR "labor deliveries"[Title/Abstract:~4] |
| #2 | Low Birth Weight | "Birth Weight"[Mesh] OR "birthweight*"[Title/Abstract] OR "birth weight"[Title/Abstract:~4] OR "Fetal Weight"[Mesh] OR "fetal weight"[Title/Abstract:~4] OR "foetal weight"[Title/Abstract:~4] OR "fetus weight"[Title/Abstract:~4] OR "embryo weight"[Title/Abstract:~4] OR "small gestational"[Title/Abstract:~4] OR "low weight*"[Title/Abstract] OR "LBW"[Title/Abstract] OR "LBWs"[Title/Abstract] OR "LBWI"[Title/Abstract] OR "ELBW"[Title/Abstract] OR "ELBWI"[Title/Abstract] OR "ELBWs"[Title/Abstract] OR "VLBW"[Title/Abstract] OR "VLBWI"[Title/Abstract] OR "VLBW-I"[Title/Abstract:~1] OR "VLBWs"[Title/Abstract] OR "underweight*"[Title/Abstract] OR "weight loss*"[Title/Abstract] OR "loss weight"[Title/Abstract:~4] OR "weight reduction*"[Title/Abstract] OR "weight status*"[Title/Abstract] OR "Fetal Growth Retardation"[Mesh] OR "Fetal Growth Retardation"[Title/Abstract] OR "FGR"[Title/Abstract] |
| #3 | Health Disparities | "Health Inequities"[Mesh] OR "inequit*"[Title/Abstract] OR "equit*"[Title/Abstract] OR "inequal*"[Title/Abstract] OR "disparit*"[Title/Abstract] OR "access healthcare"[Title/Abstract:~4] OR "Social Determinants of Health"[Mesh] OR "SDOH"[Title/Abstract] OR "social determinant*"[Title/Abstract] OR "structural determinant*"[Title/Abstract] OR "financial*"[Title/Abstract] OR "low income*"[Title/Abstract] OR "poverty"[Title/Abstract] OR "Poverty"[Mesh] OR "loss income"[Title/Abstract:~4] OR "Delivery of Health Care"[Mesh] OR "delivery health care"[Title/Abstract:~4] OR "delivery healthcare"[Title/Abstract:~4] OR "delivery care"[Title/Abstract:~2] OR "delivery services"[Title/Abstract:~4] OR "healthcare system*"[Title/Abstract] OR "Maternal Nutritional Physiological Phenomena"[Mesh] OR "Malnutrition"[Mesh] OR "malnutrition"[Title/Abstract] OR "food desert*"[Title/Abstract] OR "food securit*"[Title/Abstract] OR "food insecurit*"[Title/Abstract] OR "Diet, Healthy"[Mesh] OR "diet*"[Title/Abstract] OR "nutrition*"[Title/Abstract] OR "healthy food*"[Title/Abstract] OR "Health Literacy"[Mesh] OR "literac*"[Title/Abstract] OR "Communication Barriers"[Mesh] OR "barrier*"[Title/Abstract] OR "obstacle*"[Title/Abstract] OR "second language*"[Title/Abstract] OR "English proficiency"[Title/Abstract] OR "language proficiency"[Title/Abstract] OR "Appointments and Schedules"[Mesh] OR "appointment*"[Title/Abstract] OR "well visit*"[Title/Abstract] OR "preventive medicine"[Title/Abstract] OR "preventive care"[Title/Abstract:~3] OR "Socioeconomic Factors"[Mesh] OR "socioeconomic*"[Title/Abstract] OR "socio-economic*"[Title/Abstract] OR "Transportation"[Mesh] OR "transport*"[Title/Abstract] OR "Built Environment"[Mesh] OR "environment*"[Title/Abstract] OR "socioenvironment*"[Title/Abstract] OR "ecologic*"[Title/Abstract] OR "socioecologic*"[Title/Abstract] OR "Ill-Housed Persons"[Mesh] OR "Ill-hous*"[Title/Abstract] OR "unhous*"[Title/Abstract] OR "homeless*"[Title/Abstract] OR "shelter*"[Title/Abstract] OR "Community-Based Participatory Research"[Mesh] OR "communit*"[Title/Abstract] OR "neighborhood*"[Title/Abstract] OR "Health Education"[Mesh] OR "education*"[Title/Abstract]  OR "communicat*"[Title/Abstract] OR "Cultural Competency"[Mesh] OR "cultural*"[Title/Abstract] OR "accultur*"[Title/Abstract] OR "acultur*"[Title/Abstract] OR "Health Disparate Minority and Vulnerable Populations"[Mesh] OR "Minority Health"[Mesh] OR "minorit*"[Title/Abstract] OR "race"[Title/Abstract] OR "Prejudice"[Mesh] OR "prejudice*"[Title/Abstract] OR "racism*"[Title/Abstract] OR "racial*"[Title/Abstract] OR "multiracial*"[Title/Abstract] OR "implicit bias"[Title/Abstract:~4] OR "microaggression*"[Title/Abstract] OR "micro-aggression*"[Title/Abstract] OR "microinsult*"[Title/Abstract] OR "micro insult*"[Title/Abstract] OR "microinvalidat*"[Title/Abstract] OR "microassault*"[Title/Abstract] OR "Social Discrimination"[Mesh] OR "discriminat*"[Title/Abstract] OR "Social Stigma"[Mesh] OR "stigma*"[Title/Abstract] OR "Ethnicity"[Mesh] OR "ethnic*"[Title/Abstract] OR "multiethnic*"[Title/Abstract] OR "vulnerab*"[Title/Abstract] OR "disadvantage*"[Title/Abstract] OR "underserve*"[Title/Abstract] OR "under-serve*"[Title/Abstract] OR "underrepresent*"[Title/Abstract] OR "under-represent*"[Title/Abstract] OR "marginaliz*"[Title/Abstract] OR "Emigrants and Immigrants"[Mesh] OR "emigrant*"[Title/Abstract] OR "immigrant*"[Title/Abstract] OR "refugee*"[Title/Abstract] OR "asylum seeker*"[Title/Abstract] OR "undocumented"[Title/Abstract] OR "sociodemographic*"[Title/Abstract] OR "socio demographic*"[Title/Abstract] OR "African American*"[Title/Abstract] OR "Black"[Title/Abstract] OR "Blacks"[Title/Abstract] OR "Hispanic*"[Title/Abstract] OR "Latino*"[Title/Abstract] OR "Latina*"[Title/Abstract] OR "Latinx"[Title/Abstract] OR "Latine"[Title/Abstract] OR "Asian*"[Title/Abstract] OR "Pacific Islander*"[Title/Abstract] OR "Afro-Caribbean*"[Title/Abstract] OR "AfroCaribbean*"[Title/Abstract] OR "AfroLatin*"[Title/Abstract] OR "Arab*"[Title/Abstract] OR "American Indian*"[Title/Abstract] OR "Native American*"[Title/Abstract] OR "Alaska Native*"[Title/Abstract]  OR "Native Alaskan*"[Title/Abstract] OR "Hawaiian Native*"[Title/Abstract] OR "Native Hawaiian*"[Title/Abstract] OR "Health Services, Indigenous"[Mesh] OR "Nunavut*"[Title/Abstract] OR "Aborigin**"[Title/Abstract] OR "indigene*"[Title/Abstract] OR "indigenous*"[Title/Abstract] OR "Autochtone*"[Title/Abstract] OR "Eskimo*"[Title/Abstract] OR "Esquimau*"[Title/Abstract] OR "First Nation*"[Title/Abstract] OR "First People*"[Title/Abstract] OR "Inuit*"[Title/Abstract] OR "Innuit*"[Title/Abstract] OR "Inuk*"[Title/Abstract] OR "Inupiat*"[Title/Abstract] OR "Amerind*"[Title/Abstract] OR "Original People*"[Title/Abstract] OR "Homosexuality"[Mesh] OR "homosexual*"[Title/Abstract] OR "gay"[Title/Abstract] OR "queer*"[Title/Abstract] OR "lesbian*"[Title/Abstract] OR "sexual orientation*"[Title/Abstract] OR "LGBTQ*"[Title/Abstract] OR "Transsexualism"[Mesh] OR "transexual*"[Title/Abstract] OR "transgender*"[Title/Abstract] OR "gender fluid*"[Title/Abstract] OR "gender reassign*"[Title/Abstract] OR "Bisexuality"[Mesh] OR "bisexual*"[Title/Abstract] OR "bi-sexual*"[Title/Abstract] OR "Gender Dysphoria"[Mesh] OR "dysphoria*"[Title/Abstract] OR "gender identit*"[Title/Abstract] OR "nonbinary*"[Title/Abstract] OR "non-binary*"[Title/Abstract] |
| #3 | Systematic Review | "Systematic Review" [Publication Type] OR "Systematic Reviews as Topic"[Mesh] OR "systematic review"[Filter] OR "systematic review"[Title/Abstract:~2] OR "systematic overview*"[Title/Abstract] OR "Meta-Analysis"[Publication Type] OR "Meta-Analysis as Topic"[Mesh] OR "meta analy*"[Title/Abstract] OR "metanaly*"[Title/Abstract] OR "metaanaly*"[Title/Abstract] OR "met analy*"[Title/Abstract] OR "meta synthes*"[Title/Abstract] OR  "scoping review*"[Title/Abstract] OR "scoping review"[Title/Abstract:~2] OR "rapid review*"[Title/Abstract] OR "rapid review"[Title/Abstract:~2] OR "umbrella review*"[Title/Abstract] OR "review of reviews"[Title/Abstract] |
| #4 | Limits Filters | ((#1 AND #2 AND #3 AND #4) NOT ("Animals"[Mesh] NOT ("Animals"[Mesh] AND "Humans"[Mesh]))) NOT ("Letter"[Publication Type] OR "Editorial" [Publication Type] OR "Comment"[Publication Type] OR "News"[Publication Type] OR "Retracted Publication"[Publication Type] OR "Retraction of Publication"[Publication Type] OR "retraction of publication"[Title/Abstract] OR "retraction notice"[Title] OR "retracted publication"[Title/Abstract] OR "protocol*"[Title] OR "symposium*"[Title/Abstract] OR "Congress"[Publication Type] OR "Consensus Development Conference"[Publication Type] OR "conference abstract*"[Title/Abstract] OR "conference proceeding*"[Title/Abstract] OR "conference paper*"[Title/Abstract] OR "conference review*"[Title/Abstract] OR "protocol*"[Title] OR "Guidelines as Topic"[Mesh] OR "Guideline" [Publication Type] OR "guideline*"[Title]) Filters: English, from 2019/1/1 - 2024/2/22 |

Database: Embase

Platform: Elsevier 
Date Searched: 2/22/2024 
Date Limits: 2019-2024

|  | Concept: | Search Strategy: |
| --- | --- | --- |
| #1 | Birth | 'infant'/exp OR 'immature and premature labor'/exp OR 'obstetric delivery'/exp OR 'infan*':ab,ti OR 'birth*':ab,ti OR 'baby':ab,ti OR 'babies':ab,ti OR 'born':ab,ti OR 'newborn*':ab,ti OR 'new born*':ab,ti OR 'newly born*':ab,ti OR 'prematur*':ab,ti OR 'pre-matur*':ab,ti OR 'preterm*':ab,ti OR 'neonat*':ab,ti OR 'neo-nat*':ab,ti OR 'nicu':ab,ti OR 'nicus':ab,ti OR 'premie*':ab,ti OR 'preemie*':ab,ti OR 'childbirth*':ab,ti OR 'cesarean*':ab,ti OR 'c-section*':ab,ti OR 'induced labor*':ab,ti OR ((vaginal NEAR/4 deliver*):ab,ti) OR ((labor NEAR/4 deliver*):ab,ti) |
| #2 | Low Weight | 'birth weight'/exp OR 'fetus weight'/exp OR 'intrauterine growth retardation'/exp OR 'birthweight*':ab,ti OR ((birth NEAR/4 weight):ab,ti) OR ((fetal NEAR/4 weight):ab,ti) OR ((foetal NEAR/4 weight):ab,ti) OR ((fetus NEAR/4 weight):ab,ti) OR ((embryo NEAR/4 weight):ab,ti) OR ((small NEAR/4 gestational):ab,ti) OR 'low weight*':ab,ti OR 'lbw':ab,ti OR 'lbws':ab,ti OR 'lbwi':ab,ti OR 'elbw':ab,ti OR 'elbwi':ab,ti OR 'elbws':ab,ti OR 'vlbw':ab,ti OR 'vlbwi':ab,ti OR 'vlbw-i':ab,ti OR 'vlbws':ab,ti OR 'underweight*':ab,ti OR 'weight loss*':ab,ti OR ((loss NEAR/4 weight):ab,ti) OR 'weight reduction*':ab,ti OR 'weight status*':ab,ti OR 'fetal growth retardation':ab,ti OR 'fgr':ab,ti |
| #3 | Health Disparities | 'health disparity'/exp OR 'social inequality'/exp OR 'social determinants of health'/exp OR 'poverty'/exp OR 'health care delivery'/mj OR 'maternal nutrition'/exp OR 'malnutrition'/exp OR 'healthy diet'/exp OR 'health literacy'/exp OR 'communication barrier'/exp OR 'patient attendance'/exp OR 'socioeconomic vulnerability'/exp OR 'traffic and transport'/exp OR 'built environment'/exp OR 'homeless person'/exp OR 'participatory research'/exp OR 'health education'/de OR 'cultural competence'/exp OR 'vulnerable population'/exp OR 'ethnic group'/exp OR 'minority group'/exp OR 'ancestry group'/exp OR 'prejudice'/exp OR 'social discrimination'/exp OR 'social stigma'/exp OR 'migrant'/exp OR 'minority health'/exp OR 'indigenous health care'/exp OR 'homosexuality'/exp OR 'bisexuality'/exp OR 'gender dysphoria'/exp OR 'inequit*':ab,ti OR 'equit*':ab,ti OR 'inequal*':ab,ti OR 'disparit*':ab,ti OR ((access NEAR/4 healthcare):ab,ti) OR 'sdoh':ab,ti OR 'social determinant*':ab,ti OR 'structural determinant*':ab,ti OR 'financial*':ab,ti OR 'low income*':ab,ti OR 'poverty':ab,ti OR ((loss NEAR/4 income):ab,ti) OR ((delivery NEAR/4 'health care'):ab,ti) OR ((delivery NEAR/4 healthcare):ab,ti) OR ((delivery NEAR/2 care):ab,ti) OR ((delivery NEAR/4 services):ab,ti) OR 'healthcare system*':ab,ti OR 'malnutrition':ab,ti OR 'food desert*':ab,ti OR 'food securit*':ab,ti OR 'food insecurit*':ab,ti OR 'diet*':ab,ti OR 'nutrition*':ab,ti OR 'healthy food*':ab,ti OR 'literac*':ab,ti OR 'barrier*':ab,ti OR 'obstacle*':ab,ti OR 'second language*':ab,ti OR 'english proficiency':ab,ti OR 'language proficiency':ab,ti OR 'appointment*':ab,ti OR 'well visit*':ab,ti OR 'preventive medicine':ab,ti OR ((preventive NEAR/3 care):ab,ti) OR 'socioeconomic*':ab,ti OR 'socio-economic*':ab,ti OR 'transport*':ab,ti OR 'environment*':ab,ti OR 'socioenvironment*':ab,ti OR 'ecologic*':ab,ti OR 'socioecologic*':ab,ti OR 'ill-hous*':ab,ti OR 'unhous*':ab,ti OR 'homeless*':ab,ti OR 'shelter*':ab,ti OR 'communit*':ab,ti OR 'neighborhood*':ab,ti OR 'education*':ab,ti OR 'communicat*':ab,ti OR 'cultural*':ab,ti OR 'accultur*':ab,ti OR 'acultur*':ab,ti OR 'minorit*':ab,ti OR 'race':ab,ti OR 'prejudice*':ab,ti OR 'racism*':ab,ti OR 'racial*':ab,ti OR 'multiracial*':ab,ti OR ((implicit NEAR/4 bias):ab,ti) OR 'microaggression*':ab,ti OR 'micro-aggression*':ab,ti OR 'microinsult*':ab,ti OR 'micro insult*':ab,ti OR 'microinvalidat*':ab,ti OR 'microassault*':ab,ti OR 'discriminat*':ab,ti OR 'stigma*':ab,ti OR 'ethnic*':ab,ti OR 'multiethnic*':ab,ti OR 'vulnerab*':ab,ti OR 'disadvantage*':ab,ti OR 'underserve*':ab,ti OR 'under-serve*':ab,ti OR 'underrepresent*':ab,ti OR 'under-represent*':ab,ti OR 'marginaliz*':ab,ti OR 'emigrant*':ab,ti OR 'immigrant*':ab,ti OR 'refugee*':ab,ti OR 'asylum seeker*':ab,ti OR 'undocumented':ab,ti OR 'sociodemographic*':ab,ti OR 'socio demographic*':ab,ti OR 'african american*':ab,ti OR 'black':ab,ti OR 'blacks':ab,ti OR 'hispanic*':ab,ti OR 'latino*':ab,ti OR 'latina*':ab,ti OR 'latinx':ab,ti OR 'latine':ab,ti OR 'asian*':ab,ti OR 'pacific islander*':ab,ti OR 'afro-caribbean*':ab,ti OR 'afrocaribbean*':ab,ti OR 'afrolatin*':ab,ti OR 'arab*':ab,ti OR 'american indian*':ab,ti OR 'native american*':ab,ti OR 'alaska native*':ab,ti OR 'native alaskan*':ab,ti OR 'hawaiian native*':ab,ti OR 'native hawaiian*':ab,ti OR 'nunavut*':ab,ti OR 'aborigin**':ab,ti OR 'indigene*':ab,ti OR 'indigenous*':ab,ti OR 'autochtone*':ab,ti OR 'eskimo*':ab,ti OR 'esquimau*':ab,ti OR 'first nation*':ab,ti OR 'first people*':ab,ti OR 'inuit*':ab,ti OR 'innuit*':ab,ti OR 'inuk*':ab,ti OR 'inupiat*':ab,ti OR 'amerind*':ab,ti OR 'original people*':ab,ti OR 'homosexual*':ab,ti OR 'gay':ab,ti OR 'queer*':ab,ti OR 'lesbian*':ab,ti OR 'sexual orientation*':ab,ti OR 'lgbtq*':ab,ti OR 'transexual*':ab,ti OR 'transgender*':ab,ti OR 'gender fluid*':ab,ti OR 'gender reassign*':ab,ti OR 'bisexual*':ab,ti OR 'bi-sexual*':ab,ti OR 'dysphoria*':ab,ti OR 'gender identit*':ab,ti OR 'nonbinary*':ab,ti OR 'non-binary*':ab,ti |
| #3 | Systematic Review | 'systematic review'/exp OR 'systematic review (topic)'/exp OR 'meta analysis'/exp OR 'meta analysis (topic)'/exp OR 'scoping review'/exp OR 'rapid review'/exp OR 'umbrella review'/exp OR ((systematic NEAR/2 review):ab,ti) OR 'systematic overview*':ab,ti OR 'meta analy*':ab,ti OR 'metanaly*':ab,ti OR 'metaanaly*':ab,ti OR 'met analy*':ab,ti OR 'meta synthes*':ab,ti OR 'scoping review*':ab,ti OR ((scoping NEAR/2 review):ab,ti) OR 'rapid review*':ab,ti OR ((rapid NEAR/2 review):ab,ti) OR 'umbrella review*':ab,ti OR 'review of reviews':ab,ti |
| #4 | Limits Filters | #1 AND #2 AND #3 AND #4 AND [english]/lim AND [2019-2024]/py NOT ([animals]/lim NOT ([animals]/lim AND [humans]/lim)) NOT ([conference abstract]/lim OR [conference paper]/lim OR [conference review]/lim OR 'conference abstract*':ab,ti OR 'conference proceeding*':ab,ti OR 'conference paper*':ab,ti OR [editorial]/lim OR 'retraction notice'/exp OR 'retraction of publication':ab,ti OR 'retraction notice':ti OR 'retracted publication':ab,ti OR [letter]/lim OR [note]/lim OR 'protocol*':ti OR 'practice guideline'/exp OR 'guideline*':ti) |
