# Supplementary material for: Enabling discovery of the social determinants of health: using a specialized lens to see beyond the surface
Source: J Med Libr Assoc. 2025 Aug 1;113(3):204–22. doi: 10.5195/jmla.2025.2186 (PMC12369968; doi:10.5195/jmla.2025.2186)
Supplement: Supplementary file 2 — Appendix B: Study Characteristics [file jmla-113-3-204-s02.docx]

**Appendix B: Study Characteristics**

**Table 1 - Study Characteristics**

| Author, Year | Population Characteristics | Study Design | Country | Study Aim | Summary of Findings |
| --- | --- | --- | --- | --- | --- |
| Ahrens, 2019 [16] | Postpartum women of reproductive age with at least one livebirth or whose last delivery was a stillbirth. | Systematic Review | Amsterdam, Australia, Canada, Denmark, Poland, Scotland, Sweden, USA | Associations between short interpregnancy intervals and adverse perinatal outcomes in high-resource settings. | Interpregnancy interval < 6 months since last livebirth associated with risk for preterm birth, small for gestational age and infant death. |
| Amjad, 2019 [17] | Adolescent mothers who gave birth before the age of 20 years. | Systematic Review Meta-Analysis | Australia, Brazil, Colombia,  Israel, Malaysia, Nigeria,  Senegal, Spain, Trinidad, UK, USA | Association between SDoH and maternal and  birth outcomes in adolescent mothers. | Different definitions of SDoH made direct comparison and pooling of data difficult. Adverse pregnancy outcomes experienced by adolescent mothers are a result of social deprivation young mothers' encounter. |
| Atherton, 2023 [18] | Patients who were referred to newborn hearing screening. | Systematic Review Meta-Analysis | Brazil, China, Nigeria, South Africa, USA | Identify and assess infant and maternal sociodemographic factors associated with loss of follow-up. | Sociodemographic characteristics of infants and their mothers may be at high risk for nonadherence to early hearing detection and follow-up compliance. |
| Behboudi-Gandevani, 2022 [19] | Immigrant women who crossed international border (refugees, asylum seekers, illegal and undocumented immigrants, and economic and transient immigrants). | Systematic Review Meta-Analysis | Argentine, Asia, Australia, Austria, Belgium, Canada Chile, Denmark, Finland,  France, Germany, Greece, Israel, Italy, Jordan, Pakistan, Portugal, Korea, Lebanon, New Zealand, The Netherlands, Norway, South America, Spain, Sweden, Switzerland, Taiwan, Turkey, UK, USA | Investigate the risk of adverse maternal and neonatal outcomes in immigrant women compared with native-origin women in the host country. | Perinatal health of immigrants’ risk of adverse maternal and neonatal outcomes. |
| Bekkar, 2020 [20] | Pregnant women in U.S. populations. | Systematic Review | USA | Prenatal exposure to fine particulate matter (PM2.5), ozone, and heat, and the association of these factors with preterm birth, low birth weight, and stillbirth. | Association of air pollutant and heat exposure with birth outcomes. |
| Bellerose, 2022 [21] | Preconception, pregnancy, and postpartum women in U.S. population. | Systematic Review | USA | Effects of the Affordable Care Act Medicaid expansion on perinatal health insurance coverage, health care use, and health outcomes overall (primary objective) and by race and ethnicity (secondary objective). | Medicaid expansion impact an increased perinatal Medicaid coverage and decreased perinatal uninsurance and private coverage. |
| Boccia, 2023 [22] | Childrens’ first 1,000 days of life. | Systematic Review | Canada, UK, USA | Impact of interventions able to modify  the effect of early-life socioeconomic stressors during the first 1,000 days of life. | Income support policies delivered in the first 1,000 days of life are able to improve important life-course risk factors and child health outcomes. |
| Bowden, 2023 [23] | Women from high-income countries, known to have an increased risk of maternal mortality and severe maternal morbidity accessing antenatal, intrapartum, or postpartum care. | Systematic Review Meta-Analysis | USA | Improve care-seeking in pregnancy, birthing, and the postnatal period (up to 6 weeks), and improve clinical outcomes, for women living with vulnerabilities, and their babies, in high-income countries. | Utilization of health services, attendance at antenatal classes and postnatal visits by 6–8 weeks. |
| Cai, 2020 [24] | Pregnant women (any trimester) who engaged in paid work. | Systematic Review Meta-Analysis | Canada, China, Denmark, Egypt, Finland, France, Germany, Guatemala, Iran, Ireland, Italy,  Lithuania, Mexico,  Netherlands, Poland, Portugal, South Korea, Spain, Sweden, Thailand, UK,  USA | Evaluate the impact of physically demanding occupational activities on Maternal and fetal health outcomes. | Occupational activities heavy lifting, prolonged, standing, prolonged walking, prolonged Bending, and heavy physical workload increase the risks of important adverse pregnancy outcomes, such as preterm delivery (PTD), low birth weight LBW, small for gestational age (SGA), and preeclampsia. |
| Chang, 2022 [25] | Migrant children aged 0–18 years. Both internal and international migrations. | Systematic Review Meta-Analysis | Australia, Belgium, Canada, China, Denmark, Finland, Germany, India,  Italy, Netherlands,  New Zealand, Portugal, Russia, Spain, Sweden, Switzerland, Taiwan, USA | Evaluate the impact of migration on major health indicators, including children's birth outcome, nutrition, physical health, mental health, death, and substance use. | Migrant children overall worse health outcomes than non-migrant children (malnutrition, oral disease, depression, and death). |
| Chersich, 2020 [26] | Preterm birth live birth before 37  completed weeks of gestation. | Systematic Review Meta-Analysis | Australia, China, European Union, New Zealand,  North America, Sub-Saharan Africa | Exposure to high temperatures in pregnancy is associated with increased risk for preterm birth, low birth weight, and stillbirth. | Heat exposure increases the likelihood of adverse pregnancy outcome. |
| Ciulei, 2023 [27] | Pregnant women. | Systematic Review Meta-Analysis | Bangladesh, Burkina Faso,  Chile, Colombia, Gambia, Ghana, India, Indonesia,  Iran, Malawi, South Africa, Thailand, UK, USA | Define two common types of nutritious supplemental foods used in pregnancy, balanced energy-protein supplements and lipid-based nutrient supplements (LNS). | LNS is compared with iron and folic acid in improving maternal and infant outcomes. |
| Crawford, 2022 [28] | Childbearing women in USA. | Scoping Review | USA | Microaggression as an experience of racism and its influence on perinatal health outcomes. | Instances of microaggressions, such as feeling discriminated against, ignored, or dismissed, experienced by Black women can negatively influence perinatal health outcomes in childbearing women. |
| Di Tosto, 2021 [29] | Pregnant individuals at any point during pregnancy or up to 6 weeks postpartum. | Systematic Review | USA | Housing instability during pregnancy and adverse pregnancy outcomes and perinatal healthcare utilization. | A relationship between homelessness or housing instability and low birthweight or preterm  birth. |
| Dzekem, 2024 [30] | Pregnant women across all races. | Systematic Review | USA | Explore the importance of race as a risk factor for air pollution-related poor pregnancy outcomes. | Impact of air pollution on LBW, PTD, SGA and stillbirths within racial and ethnic minorities. |
| East, 2019 [31] | Pregnant women at risk of having preterm or growth restricted babies, or both. | Systematic Review Meta-Analysis | Africa, Australia, France, Ireland, Latin America,  South Africa, UK, USA | Programmes offering additional social support (emotional, instrumental/tangible and informational) compared with routine care, for pregnant women believed to be at high risk for giving birth to babies that are either preterm (less than 37 weeks' gestation) or weigh less than 2500 g, or both, at birth. | Programmes offering additional social support for at-risk pregnant women may slightly reduce the number of babies born with a birthweight. |
| Heo 2019 [32] | Pregnant women risk of low birth weight, small for gestational age, stillbirth. | Systematic Review | Belgium, Canada, China, France, Italy, Norway, South Korea,  UK, USA | Maternal risk factors, including socioeconomic status (SES), demographics, combined with particulate matter (PM) exposure during pregnancy the risk of adverse birth outcomes including LBW, SGA, PTB, and stillbirth. | Association between maternal exposure to air pollution health disparities risk factors related to adverse birth outcomes. |
| Karger, 2022 [33] | Indigenous population. | Systematic Review | Australia, Sweden, USA | Identify interventions that aimed to reduce the incidence of preterm birth and low birthweight births in Indigenous  and CALD mothers and infants and examine which levels of the social-ecological model were addressed. | Culturally appropriate interventions for reducing low birthweight, and prematurity birth outcomes for marginalized indigenous women. |
| Khan, 2023 [34] | Ethnic minorities, indigenous people. | Systematic Review | Australia, Canada, Chile,  Hong Kong, UK, USA | Targeted health and social care interventions in HICs to reduce health inequalities experienced by disproportionately at-risk women and infants. | Multi-interventional approaches targeted for at-risk populations, in particular combining midwifery models of care with community-centered approaches, to enhance accessibility, earlier engagement, and increased attendance. |
| Koivu, 2023 [35] | Pregnant females, irrespective of gestational age. | Systematic Review | Australia, Denmark,  France, Ireland, Netherlands, UK, USA | Interventions addressing harmful behaviors, psychosocial risks, and unfavorable socioeconomic factors in pregnancy to reduce LBW and related adverse birth outcomes. | Professionally provided psychosocial support during pregnancy in general and specifically as a means to reduce smoking can potentially contribute to improved newborn health. |
| Larrabee  Sonderlund, 2021 [36] | Pregnant women and birth complications. | Systematic Review | Germany, New Zealand, USA | Relationship between maternal experiences of interpersonal discrimination and adverse birth outcomes in minority populations. | maternal experiences of interpersonal discrimination have grave physical implications that may extend beyond women’s individual health to negatively affect their pregnancy and offspring. |
| Manzo, 2024 [37] | Active-duty servicewomen or women veterans. | Scoping Review | USA | Examine the extent to which military trauma exposures impact the pregnancy outcomes of active-duty servicewomen and women veterans. | U.S. active-duty servicewomen or women veterans' exposure to military trauma and the impact on pregnancy outcome. |
| Montoya-Williams, 2021 [38] | Hispanic/Latinx pregnant women. | Scoping Review | USA | Hispanic/Latinx perinatal outcomes over the past 2 decades and place these findings within the context of the overarching "Healthy Immigrant" paradox. | Heterogeneity of outcomes within women grouped together under a single Latina(o)(x) or Hispanic category showing levels of acculturation provide granularity to understand outcomes within this single ethnic group. |
| Nelson, 2022 [39] | Unintended pregnancy with maternal and infant health outcomes occurring during pregnancy and post-partum. | Systematic Review Meta-Analysis | Asia, Europe, North America | Estimate associations of unintended pregnancy with key maternal and infant health outcomes during pregnancy and postpartum relevant to clinical practice and public health in the United States. | Unintended pregnancy, compared with intended pregnancy, was significantly associated with adverse maternal and infant outcomes. |
| Novillo-Del-Alamo, 2023 [40] | Preterm births, small for gestational age, low birth weight stillbirth, low 5-min Apgar scores. | Systematic Review | Australia, Brazil, Canada, France, Germany, New Zealand, Netherlands, Spain, Sweden, UK, USA | Assess if women living in deprived areas have worse perinatal outcomes compared with mothers residing in high-income areas. | Deprived areas are associated with adverse perinatal outcomes, such as SGA, PTB, and stillbirth. |
| Pereira, 2022 [41] | Racism manifestation perceived by women of any age in an obstetric scenario. | Systematic Review | USA | Assess the impact of racism or racial discrimination within obstetric outcomes, considering that in obstetrics, the effect of racism may lead to racial disparities that involve both the woman and the child. | Perceived racism or racial discrimination  was negatively associated with maternal and neonatal outcomes. |
| Sheikh, 2022 [42] | Neonatal death, stillbirth, preterm birth, and small-for-gestational-age babies. | Systematic Review Meta-Analysis | Canada, Multi-Country,  Netherlands, Northern, Western and Southern Europe, UK, USA | Quantify the effects of race and ethnicity  in women from underserved groups in high-income and upper-middle-income countries on neonatal deaths and stillbirths. | Racial or ethnic groups (White, Black, south Asian, Hispanic, or other). |
| Simonovich, 2020 [43] | Age of target population 0–5 years in developed countries. | Systematic Review | USA and other developed nations | Physiological health outcomes associated with food insecurity during early childhood among children aged 0–5 years in developed countries. | Food insecurity and physiological health outcomes within this vulnerable group. |
| Syed, 2022 [44] | Live birth (full term or preterm birth), stillbirth, spontaneous abortion miscarriage, PROM (premature rupture membrane), birthweight, congenital anomalies as variables of interest. | Scoping Review | Australia, East Asia, Eastern Europe, Northern America, Northern Europe, South America, Southern Europe, West Africa, Western Asia, Western Europe, | Identify the major adverse pregnancy outcomes examined in the context of ambient gestational heat exposure, with emphasis on study design, gestational windows of sensitivity, and characterization of environmental temperatures. | Gestational heat exposure and adverse pregnancy outcomes. |
| Thayamballi, 2021 [45] | Birthweight; LBW; LBW sex-specific reference as SGA; death, or loss of a baby before or during delivery; stillbirth; and structural or functional changes present at birth as birth defects. | Systematic Review  Meta-Analysis | USA | Risk of adverse birth outcomes due to PM exposures among subpopulations and investigate whether any particular population is more vulnerable. | Effect modification by maternal race and ethnicity and educational attainment for the associations between PM exposures and birth outcomes in the United States. |
| van Daalen, 2022 [46] | Pregnant women, including PTB, LBW, and hypertensive disorder of pregnancy (HDP). | Systematic Review  Meta-Analysis | Australia, Germany, New Zealand, Serbia, USA | Association between perceived racial discrimination and adverse pregnancy outcomes. | Racial discrimination adversely impacts pregnancy outcomes, with the greatest evidence found for PTB. |

# **References**

16. Ahrens KA, Nelson H, Stidd RL, Moskosky S, Hutcheon JA. Short interpregnancy intervals and adverse perinatal outcomes in high-resource settings: An updated systematic review. Paediatr Perinat Epidemiol. 2019;33(1):O25-o47. doi: 10.1111/ppe.12503.

17. Amjad S, MacDonald I, Chambers T, Osornio-Vargas A, Chandra S, Voaklander D, et al. Social determinants of health and adverse maternal and birth outcomes in adolescent pregnancies: A systematic review and meta-analysis. Paediatr Perinat Epidemiol. 2019;33(1):88-99. doi: 10.1111/ppe.12529.

18. Atherton KM, Poupore NS, Clemmens CS, Nietert PJ, Pecha PP. Sociodemographic Factors Affecting Loss to Follow-Up After Newborn Hearing Screening: A Systematic Review and Meta-Analysis. Otolaryngol Head Neck Surg. 2023;168(6):1289-300. doi: 10.1002/ohn.221.

19. Behboudi-Gandevani S, Bidhendi-Yarandi R, Panahi MH, Mardani A, Paal P, Prinds C, et al. Adverse Pregnancy Outcomes and International Immigration Status: A Systematic Review and Meta-analysis. Ann Glob Health. 2022;88(1):44. doi: 10.5334/aogh.3591.

20. Bekkar B, Pacheco S, Basu R, DeNicola N. Association of Air Pollution and Heat Exposure With Preterm Birth, Low Birth Weight, and Stillbirth in the US: A Systematic Review. JAMA Netw Open. 2020;3(6):e208243. doi: 10.1001/jamanetworkopen.2020.8243.

21. Bellerose M, Collin L, Daw JR. The ACA Medicaid Expansion And Perinatal Insurance, Health Care Use, And Health Outcomes: A Systematic Review. Health Aff (Millwood). 2022;41(1):60-8. doi: 10.1377/hlthaff.2021.01150.

22. Boccia D, Maritano S, Pizzi C, Richiardi MG, Lioret S, Richiardi L. The impact of income-support interventions on life course risk factors and health outcomes during childhood: a systematic review in high income countries. BMC Public Health. 2023;23(1):744. doi: 10.1186/s12889-023-15595-x.

23. Bowden ER, Chang AB, McCallum GB. Interventions to improve enablers and/or overcome barriers to seeking care during pregnancy, birthing and postnatal period for women living with vulnerabilities in high-income countries: A systematic review and meta-analysis. Midwifery. 2023;121:103674. doi: 10.1016/j.midw.2023.103674.

24. Cai C, Vandermeer B, Khurana R, Nerenberg K, Featherstone R, Sebastianski M, et al. The impact of occupational activities during pregnancy on pregnancy outcomes: a systematic review and metaanalysis. Am J Obstet Gynecol. 2020;222(3):224-38. doi: 10.1016/j.ajog.2019.08.059.

25. Chang R, Li C, Qi H, Zhang Y, Zhang J. Birth and Health Outcomes of Children Migrating With Parents: A Systematic Review and Meta-Analysis. Frontiers in Pediatrics. 2022;10. doi: 10.3389/fped.2022.810150.

26. Chersich MF, Pham MD, Areal A, Haghighi MM, Manyuchi A, Swift CP, et al. Associations between high temperatures in pregnancy and risk of preterm birth, low birth weight, and stillbirths: systematic review and meta-analysis. Bmj. 2020;371:m3811. doi: 10.1136/bmj.m3811.

27. Ciulei MA, Smith ER, Perumal N, Jakazi CS, Sudfeld CR, Gernand AD. Nutritious Supplemental Foods for Pregnant Women from Food Insecure Settings: Types, Nutritional Composition, and Relationships to Health Outcomes. Curr Dev Nutr. 2023;7(6):100094. doi: 10.1016/j.cdnut.2023.100094.

28. Crawford AD, Darilek U, McGlothen-Bell K, Gill SL, Lopez E, Cleveland L. Scoping Review of Microaggression as an Experience of Racism and Perinatal Health Outcomes. J Obstet Gynecol Neonatal Nurs. 2022;51(2):126-40. doi: 10.1016/j.jogn.2021.12.007.

29. DiTosto JD, Holder K, Soyemi E, Beestrum M, Yee LM. Housing instability and adverse perinatal outcomes: a systematic review. Am J Obstet Gynecol MFM. 2021;3(6):100477. doi: 10.1016/j.ajogmf.2021.100477.

30. Dzekem BS, Aschebrook-Kilfoy B, Olopade CO. Air Pollution and Racial Disparities in Pregnancy Outcomes in the United States: A Systematic Review. J Racial Ethn Health Disparities. 2024;11(1):535-44. doi: 10.1007/s40615-023-01539-z.

31. East CE, Biro MA, Fredericks S, Lau R. Support during pregnancy for women at increased risk of low birthweight babies. Cochrane Database Syst Rev. 2019;4(4):Cd000198. doi: 10.1002/14651858.CD000198.pub3.

32. Heo S, Fong KC, Bell ML. Risk of particulate matter on birth outcomes in relation to maternal socio-economic factors: a systematic review. Environ Res Lett. 2019;14(12). doi: 10.1088/1748-9326/ab4cd0.

33. Karger S, Bull C, Enticott J, Callander EJ. Options for improving low birthweight and prematurity birth outcomes of indigenous and culturally and linguistically diverse infants: a systematic review of the literature using the social-ecological model. BMC Pregnancy Childbirth. 2022;22(1):3. Epub 20220103. doi: 10.1186/s12884-021-04307-1.

34. Khan Z, Vowles Z, Fernandez Turienzo C, Barry Z, Brigante L, Downe S, et al. Targeted health and social care interventions for women and infants who are disproportionately impacted by health inequalities in high-income countries: a systematic review. Int J Equity Health. 2023;22(1):131. doi: 10.1186/s12939-023-01948-w.

35. Koivu AM, Näsänen-Gilmore PK, Hunter PJ, Muthiani Y, Isojärvi J, Heimonen O, et al. Antenatal interventions to address harmful behaviors and psychosocial risk factors in the prevention of low birth weight. Am J Clin Nutr. 2023;117 Suppl 2:S148-s59. doi: 10.1016/j.ajcnut.2022.11.028.

36. Larrabee Sonderlund A, Schoenthaler A, Thilsing T. The Association between Maternal Experiences of Interpersonal Discrimination and Adverse Birth Outcomes: A Systematic Review of the Evidence. Int J Environ Res Public Health. 2021;18(4). doi: 10.3390/ijerph18041465.

37. Manzo LL, Dindinger RA, Batten J, Combellick JL, Basile-Ibrahim B. The Impact of Military Trauma Exposures on Servicewomen's Pregnancy Outcomes: A Scoping Review. J Midwifery Womens Health. 2024. doi: 10.1111/jmwh.13620.

38. Montoya-Williams D, Williamson VG, Cardel M, Fuentes-Afflick E, Maldonado-Molina M, Thompson L. The Hispanic/Latinx Perinatal Paradox in the United States: A Scoping Review and Recommendations to Guide Future Research. J Immigr Minor Health. 2021;23(5):1078-91. doi: 10.1007/s10903-020-01117-z.

39. Nelson HD, Darney BG, Ahrens K, Burgess A, Jungbauer RM, Cantor A, et al. Associations of Unintended Pregnancy With Maternal and Infant Health Outcomes: A Systematic Review and Meta-analysis. Jama. 2022;328(17):1714-29. doi: 10.1001/jama.2022.19097.

40. Novillo-Del-Álamo B, Martínez-Varea A, Nieto-Tous M, Morales-Roselló J. Deprived areas and adverse perinatal outcome: a systematic review. Arch Gynecol Obstet. 2023. doi: 10.1007/s00404-023-07300-5.

41. Pereira GMV, Pimentel VM, Surita FG, Silva AD, Brito LGO. Perceived racism or racial discrimination and the risk of adverse obstetric outcomes: a systematic review. Sao Paulo Med J. 2022;140(5):705-18. doi: 10.1590/1516-3180.2021.0505.R1.07042022.

42. Sheikh J, Allotey J, Kew T, Fernández-Félix BM, Zamora J, Khalil A, et al. Effects of race and ethnicity on perinatal outcomes in high-income and upper-middle-income countries: an individual participant data meta-analysis of 2 198 655 pregnancies. Lancet. 2022;400(10368):2049-62. doi: 10.1016/s0140-6736(22)01191-6.

43. Simonovich SD, Pineros-Leano M, Ali A, Awosika O, Herman A, Withington MHC, et al. A systematic review examining the relationship between food insecurity and early childhood physiological health outcomes. Transl Behav Med. 2020;10(5):1086-97. doi: EHDI guidelines

44. Syed S, O'Sullivan TL, Phillips KP. Extreme Heat and Pregnancy Outcomes: A Scoping Review of the Epidemiological Evidence. Int J Environ Res Public Health. 2022;19(4). doi: 10.3390/ijerph19042412.

45. Thayamballi N, Habiba S, Laribi O, Ebisu K. Impact of Maternal Demographic and Socioeconomic Factors on the Association Between Particulate Matter and Adverse Birth Outcomes: a Systematic Review and Meta-analysis. J Racial Ethn Health Disparities. 2021;8(3):743-55. doi: 10.1007/s40615-020-00835-2.

46. van Daalen KR, Kaiser J, Kebede S, Cipriano G, Maimouni H, Olumese E, et al. Racial discrimination and adverse pregnancy outcomes: a systematic review and meta-analysis. BMJ Glob Health. 2022;7(8). doi: 10.1136/bmjgh-2022-009227.
