# Supplementary material for: Enabling discovery of the social determinants of health: using a specialized lens to see beyond the surface
Source: J Med Libr Assoc. 2025 Aug 1;113(3):204–22. doi: 10.5195/jmla.2025.2186 (PMC12369968; doi:10.5195/jmla.2025.2186)
Supplement: Supplementary file 3 — Appendix C: Qualitative Assessment of Keywords Used [file jmla-113-3-204-s03.docx]

**Appendix C: Figures 1 and 2**

**Qualitative Assessment of Keywords Used (VOSviewer Analysis Keyterm Co-occurrence)**

**Figure 1 - Two-term analysis of occurrences of a keyword**


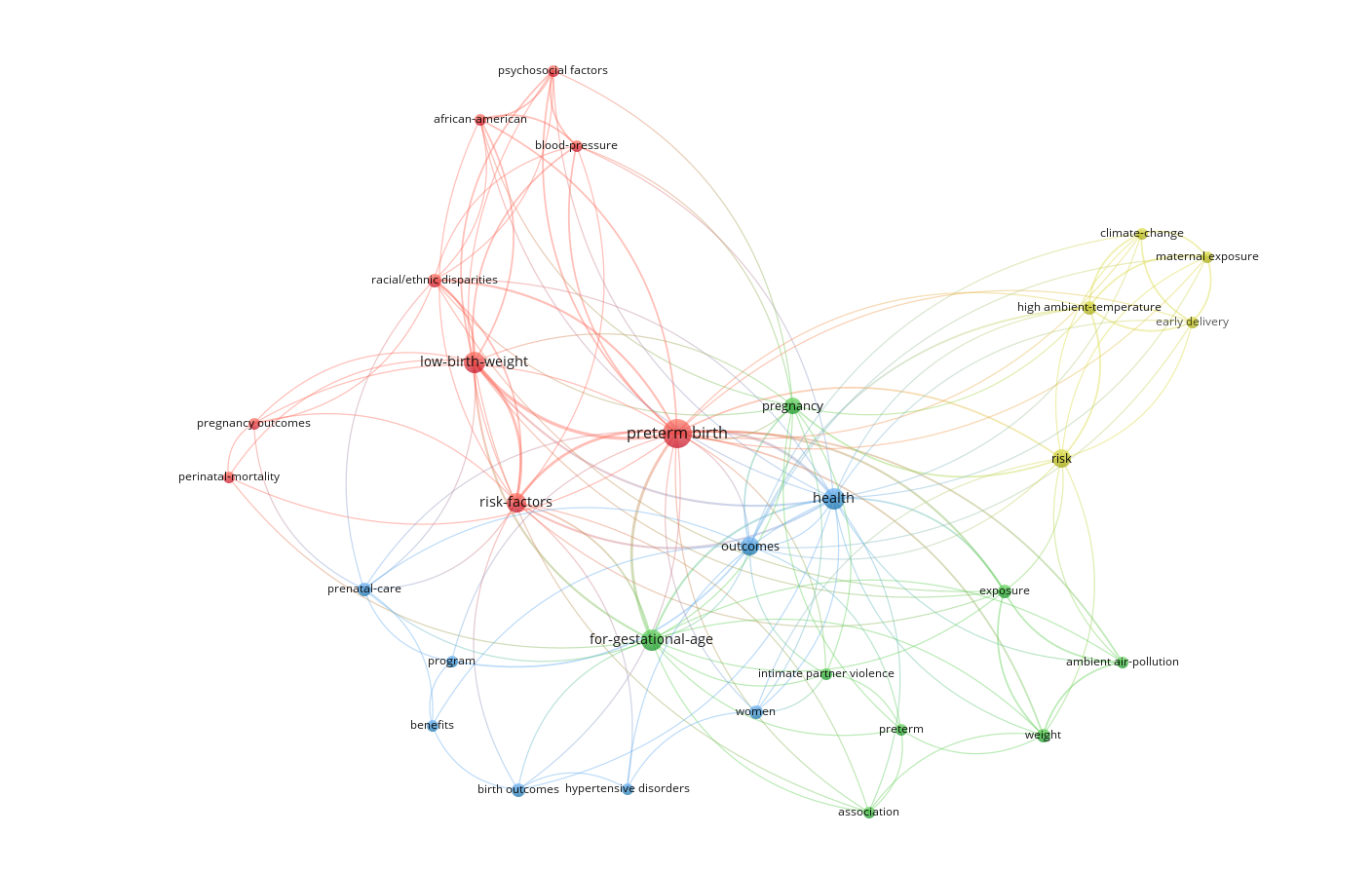


***Figure 2 - Three terms occurrences of a keyword***

**
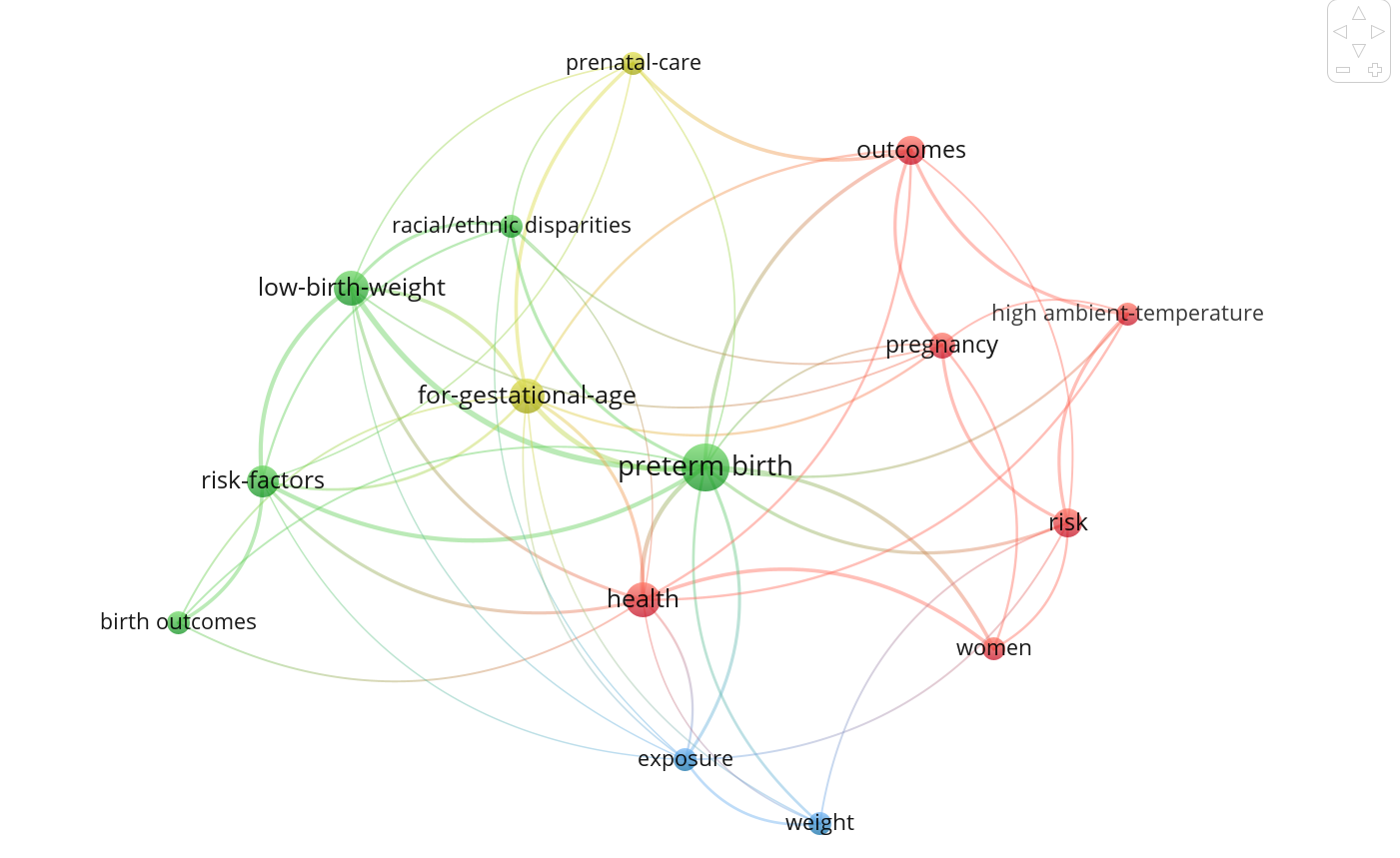
**
