# Supplementary material for: Enabling discovery of the social determinants of health: using a specialized lens to see beyond the surface
Source: J Med Libr Assoc. 2025 Aug 1;113(3):204–22. doi: 10.5195/jmla.2025.2186 (PMC12369968; doi:10.5195/jmla.2025.2186)

**Appendix D: Figures 1 - 5**

*Figures 1 – 5 -* SDoH Domains and Concepts

**Qualitative Codes (SDoH from NIMHD)**

The following images represent the qualitative assessment results from reviewing the 31 full-text articles. Images are divided by the SDoH Domains.

The “Files” column lists the number of studies coded to a SDoH concept. The Ref (References) column indicates the number of phrases identified for the corresponding concept. As themes emerged from the 31 studies, totals for each of the SDoH concepts continued to tally, therefore the image provides a visual representation of the frequency of themes within each domain.

Concepts shown toward the bottom of each domain have a higher number of concepts found, indicating they are the more predominant themes within the literature.

Figure 1 - Behavioral


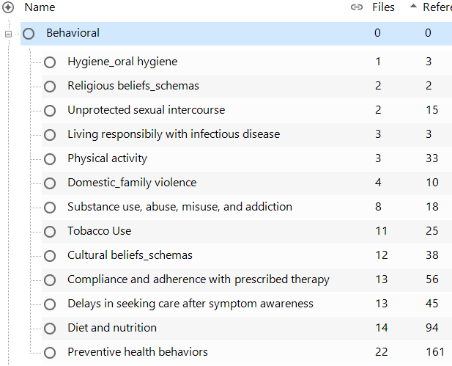


Figure 2 - Biological


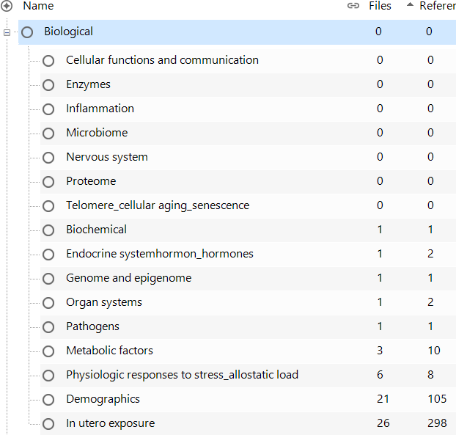


Figure 3 - Clinical Events and Health Care Systems


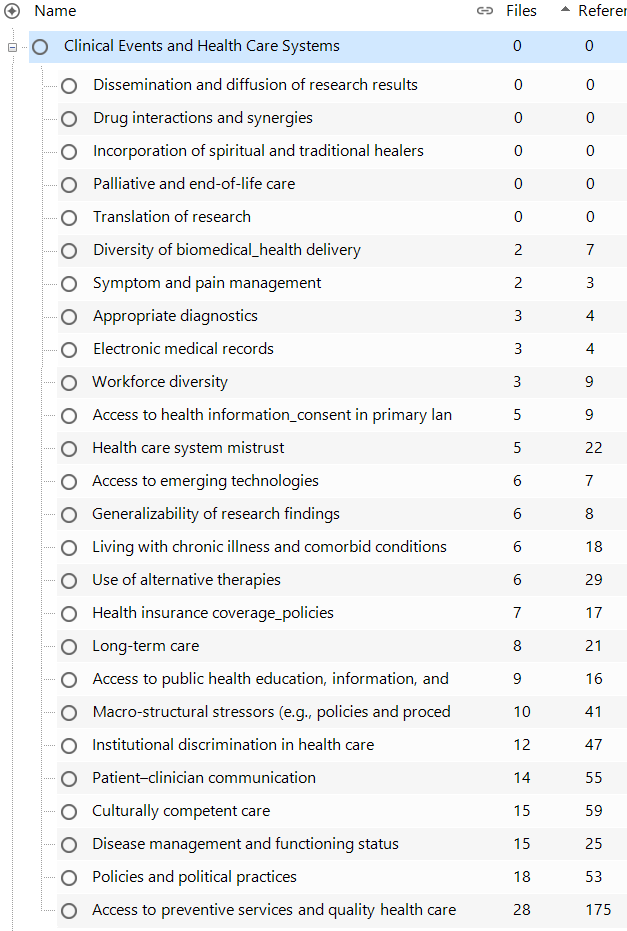


Figure 4 - Physical Environment


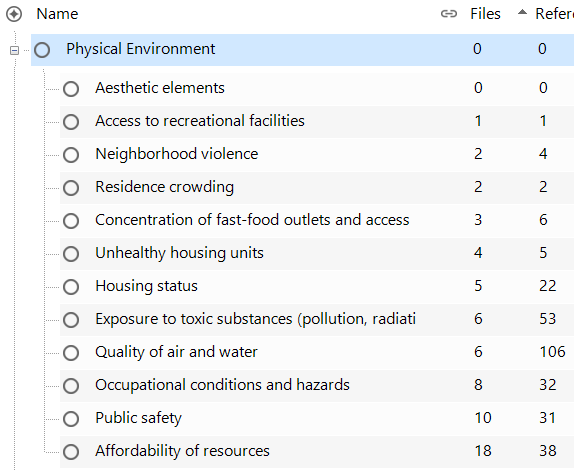


Figure 5 - Sociocultural Environment


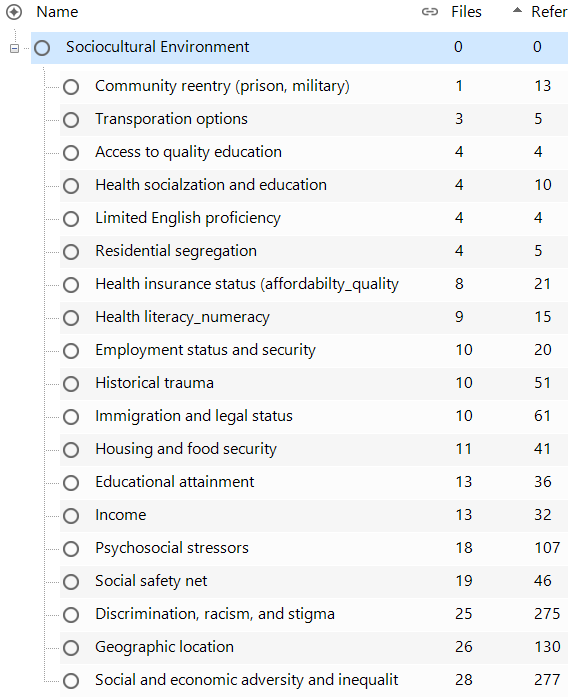

Supplement: Supplementary file 4 — Appendix D: Qualitative Code SDoH Concepts [file jmla-113-3-204-s04.docx]
