# Supplementary material for: Enabling discovery of the social determinants of health: using a specialized lens to see beyond the surface
Source: J Med Libr Assoc. 2025 Aug 1;113(3):204–22. doi: 10.5195/jmla.2025.2186 (PMC12369968; doi:10.5195/jmla.2025.2186)
Supplement: Supplementary file 5 — Appendix E: Tables 6a – 6b SDoH Concepts Emerged from Coding [file jmla-113-3-204-s05.docx]

# **Appendix E: Tables 1 and 2**

**Table 1 - Primary SDoH Coded Concepts**

| **BIOLOGICAL**  **In utero exposure*—298 coded references***  Bekkar, 2020[1]  “Studies across diverse US populations were identified that reported an association of PM2.5, ozone, and heat exposure with the adverse obstetrical outcomes of preterm birth, low birth weight at term, and stillbirth.” |
| --- |
| **SOCIOCULTURAL ENVIRONMENT**  **Discrimination, racism, and stigma*—275 coded references***  Amjad, 2019[2]  “Previous research suggests that race‐based differences in adverse pregnancy outcomes are the result of systemic racism faced by African American mothers. Race as a social institution perpetuates social stratification and ostracization which can potentially result in systematic exclusion of populations of adolescent mothers, putting them at a higher risk of poor maternal and birth outcomes. Maternal race has been suggested to operate through social hierarchies and the resulting differential access to economic structures, legal systems, education, employment opportunities, health care services by African American mothers.”  **Social and economic adversity and inequality*—277 coded references***  Dzekem, 2024[3]  “Trends in air pollution and racial disparities suggest worse exposure in people of color (POC) when compared to non-Hispanic whites (NHW). … They concluded that for all years and pollutants, the racial/ethnic group with the highest national average exposure was a racial/ ethnic minority group (NHB), with a national mean air pollution exposure higher for all three racial/ethnic minorities than for NHW.” |
| **CLINICAL EVENTS AND HEALTH CARE SYSTEM**  **Access to preventative services and quality health care*—175 coded references***  Bowden, 2023[4]  “These data are important as engagement with health services during the antenatal period provides opportunities for detection of complications, focused health education, emergency preparedness and identification of the onset of labour, thus reducing the risk of perinatal and maternal complications, including severe maternal morbidity and mortality.” |

**Table 2 - SDoH Coded Concepts**

| **Domain/Concept** | **Author/Quote** |
| --- | --- |
| **BEHAVIORAL** | |
| Preventive health behaviors | **Khan, 2023 [5]**  Secondary outcomes were positively associated with interventions such as earlier access to care, more than four antenatal visits coverage, mode of birth (decreased caesarean-section, and increased vaginal birth), use of postnatal contraception, increased breastfeeding rates. (1 p15) |
|  | **Atherton, 2023 [6]**  This systematic review and meta‐analysis identify signiﬁcant sociodemographic characteristics of infants and their mothers who may be at high risk not only for nonadherence with EHDI guidelines but for complete LTF after NBHS. These factors can be used to identify infants who may be at high risk for LTF and therefore provide increased counseling or services to help combat that risk. Without appropriate follow‐up, these infants will progress to school age with “unknown” reasons for NBHS failure, potentially causing detrimental learning and developmental delays. Focused efforts should be made by medical providers and policy‐makers to address these factors to ensure appropriate newborn hearing care and interventions are achieved. (2 p10) |
| Diet and nutrition | **Chang, 2022 [7]**  Our study also suggested that international migrant children had a higher risk of overweight/obesity, but the opposite result was observed among children migrating within the country. As we known, international migrants from low-middle income countries to high income countries were more likely to adopt the abovementioned westernized lifestyle and unhealthy dietary habits (e.g., high energy, sugar, and fat intake) which were the key risk of overweight/obesity. (3 p17) |
|  | **Simonovich, 2020 [8]**  Historically, FI has been predominantly measured only in very specific environments, such as federal nutrition assistance clinics and federally qualifying health centers for low-income populations. Moving forward, we encourage researchers and policymakers to advocate for the implementation of systems-wide FI screening in health care settings of all families at all encounters. (4 p1096) |
| Tobacco use | **Boccia, 2023 [9]**  Health-related behaviours were predominantly mentioned, mainly smoking, alcohol, and consumption of unhealthy foods that are unevenly distributed across different socioeconomic positions. Those behaviours can directly affect infant health, acting in particular on intrauterine growth that eventually is a key determinant of birth weight. (5 p13) |
|  | **van Daalen, 2022 [10]**  Likewise, others found that smoking mediated 13.5% of the total effect of self- reported everyday discrimination and LBW. (6 p23) |
| **BIOLOGICAL** | |
| Demographics | **Bekkar, 2020 [1]**  This review suggests that increasingly common environmental exposures exacerbated by climate change are significantly associated with serious adverse pregnancy outcomes across the US. It appears that the medical community at large and women’s health clinicians in particular should take note of the emerging data and become facile in both communicating these risks with patients and integrating them into plans for care. Moreover, physicians can adopt a more active role as patient advocates to educate elected officials entrusted with public policy and insist on effective action to stop the climate crisis. (7 p8) |
|  | **Chersich, 2020 [11]**  Estimates of the global burden of disease from heat effects on newborns could be modelled from the evidence collated in this review. These estimates are key for securing funding for reducing heat exposure for pregnant women, for demonstrating the health risks of climate change more generally, and for supporting efforts to curtail greenhouse gas emissions. Given increases in the frequency and intensity of heatwaves, the number of pregnant women exposed to these conditions worldwide, and the significant individual and societal burdens associated with preterm birth and stillbirth, research and policy initiatives to deal with these connections are a high priority. (8 p10) |
| In utero exposure | **Bekkar, 2020 [1]**  Studies across diverse US populations were identified that reported an association of PM2.5, ozone, and heat exposure with the adverse obstetrical outcomes of preterm birth, low birth weight at term, and stillbirth. (7 p7) |
|  | **Dzekem, 2024 [3]**  In a cohort of 164,905 births in Michigan, Le et al. showed that there was an association between term SGA with exposure to CO and NO2 during the first trimester of pregnancy. They also reported an association between term SGA and exposure to O3 and PM10 during the later stages of pregnancy. There was evidence of stronger associations between CO and term-SGA, NO2 and term-SGA, and SO2 and term-SGA for infants of Black mothers as compared to White mothers. (9 p540) |
| Physiologic responses of stress_allostactic load | **DiTosto, 2021 [12]**  Housing instability and homelessness were associated with increased odds of maternal readmission within 90 days (aOR, 2.7; 95% CI, 2.2–3.4). Individuals who are homeless were less likely to breastfeed their infant (homeless as reference group; aOR, 1.4; 95% CI, 1.2–1.6) and less likely to exclusively breastfeed (77.4% vs 80.8%; P<.0001) than individuals who are not homeless. Among individuals in California, housing instability was associated with a long hospital length of stay (OR, 1.6; 95% CI, 1.4–1.8) postpartum ED utilization within 90 days (aOR 2.4; 95% CI, 2.1–2.8) and 1 year (aOR, 2.7; 95% CI, 2.4–3.0), and maternal readmission within 1 year (aOR, 2.6; 95% CI, 2.2–3.0). (10 p6) |
| **Clinical Events and Health Care** | |
| Access to preventive services and quality health care | **Chang, 2022 [7]**  The limited access to health service and insurance are the most challenging barriers for this situation. Experiences of health services are often unsatisfactory for migrant children, such as diﬃculties and delay in registering with the General medical Practitioners, diﬃculties securing medical appointments and missed follow-up appointments. Studies suggests that migrant children are four times as likely to be uninsured as native children. Moreover, access to health care may also be limited by their parents’ knowledge and healthcare awareness, and language and cultural barriers. Additionally, the eﬀects of poverty on access to health insurance and healthcare appear to be the strongest. Children from a migrant household are more likely to live in poverty than children from a non-migrant household. For US migrant families, children in poorer families were nearly twice as likely to have not visited a dentist and to lack a usual source of sick care, and 50% were more likely not to have visited a doctor in the previous year. (3 p17) |
|  | **Bowden, 2023 [4]**  These data are important as engagement with health services during the antenatal period provides opportunities for detection of complications, focused health education, emergency preparedness and identiﬁcation of the onset of labour, thus reducing the risk of perinatal and maternal complications, including severe maternal morbidity and mortality. (11 p9) |
| Culturally competent care | **Crawford, 2022 [13]**  The three types of microaggression were also implicated in unequal distribution of resources, economic disadvantage, low educational attainment, redlining, unstable housing, limited access to medical advice/care, decreased quality of care, lack of cultural sensitivity in health care systems, fear of medical procedures, or fear of disclosing information to health care providers. (12 p133) |
|  | **Karger, 2022 [14]**  The interventions included in this review that targeted the organisational level of the social-ecological model had a particular focus on ensuring that the care delivered was culturally appropriate.(14, p8) …  Specifically, this reflects the need by Indigenous communities to have their infants born on the land. Being born on country connects an Indigenous person to the land and community in a deeply cultural way, and provides life-long privileges and responsibilities for both the land and people. Over a decade ago, it was accurately stated that for Indigenous women in Australia, birthing has moved “from the personal to the political as governments provide policies about what is ‘best’ for Aboriginal women and their babies”. (14, p8) |
| Health insurance coverage_policies | **Bellerose, 2022 [15]**  At each point during the perinatal period, Hispanic, Black, and Indigenous women experience higher rates of uninsurance than non-Hispanic White women.7,8 Black and Indigenous women are also two to four times more likely to die from pregnancy-related causes. (14 p61) |
|  | **Bellerose, 2022 [15]**  A difference-in-differences study in Ohio reported that Medicaid expansion was associated with a 5.1-percentage-point increase in routine postpartum visit attendance among low-income women. (14 p65) |
| Institutional discrimination in health care | **Crawford, 2022 [13]**  The most common conditions that contribute to pregnancy-related deaths are hypertension and diabetes, which may be due to factors associated with quality and access to care, underlying illness, structural racism, and implicit bias in health care. (12 p126) |
|  | **Crawford, 2022 [13]**  As such, the study by Ruiz et al. is reﬂective of other research that states that when women feel discriminated against or their health concerns are ignored (a microinvalidation) by their physicians, they are more likely to withhold information about their health, which can lead to adverse health outcomes. (12 p136) |
| Policies and political practices | **Behboudi-Gandevani, 2022 [16]**  Conflicts, human rights violations, inequality, and job opportunities may force people to immigrate with the hope to improve their quality of life. However, the increasing trends of immigration resettlement, leads to the diversity of the health status among pregnant women and new mothers in host countries. Immigrant women frequently initiate the mobility process at the childbearing age. New contexts, environments, and lifestyles may expose them to biological and psychosocial risks that tend to accentuate the situation of vulnerability. (15 p10) |
|  | **van Daalen, 2022 [10]**  These include, for example, antiracism counter marketing, values affirmation and forgiveness interventions. Some of the approaches that can be taken to reduce racial inequalities in health outlined by Williams et al include: (1) creating communities of opportunity that could minimise structural racism; (2) ensuring the emphasis on ‘health for all’ and public health approaches in healthcare systems, increasing the diversity of health professionals and ensuring that patients’ social needs are addressed as part of their management; and (3) building political will and support to counteract social and health inequities. (6 p24) |

| **PHYSICAL ENVIRONMENT** | |
| --- | --- |
| Affordability of resources | **Atherton, 2023 [6]**  In this meta‐analysis, young maternal age, education level, and minority race were deﬁned differently by the individual studies included, especially those conducted outside the United States, indicating that these factors have varying culturally deﬁned implications on behavior and access to resources. (2, p7) |
|  | **Boccia, 2023 [9]**  Some studies also mentioned the “family process” conceptual model, which postulates that the extra income provided by child benefits may improve long-run outcomes, not only through direct investments but by improving also the emotional environment in which the children grow. (5, p13)  For example, Lucas et al. concluded that the monetary value of many interventions was low, as in most studies included in their review the total increase in income to intervention families was less than US$50 per month despite the fact that many parents were compelled to work full-time. Authors questioned whether the level of income increase was sufficient to affect living conditions and – we would add – it was big enough to ensure this effect translated into a health effect. (5 p15) |
| Occupational conditions and hazards | **Cai, 2020 [17]**  The pooled estimate showed that a heavy physical workload was associated with a 79% increase in the odds of LBW compared with a light physical workload. (16 p228) |
|  | **Heo, 2019 [18]**  Of the 45 studies that examined effect modiﬁcation by maternal race/ethnicity and/or SES, the most commonly studied effect modiﬁer was race/ethnicity with 31 studies. One study used race/ethnicity of infants as a potential effect modiﬁer. The other identiﬁed effect modiﬁers were area-level integrated SES (e.g. European deprivation index) by 5 studies, mothers’ educational attainment by 17 studies, mothers’ occupation (e.g. worked as farmers versus other job workers) by 2 studies, and income or poverty level by9 studies (table 2). (17 p4) |
| Quality of air and water | **Crawford, 2022 [13]**  Other researchers examined discrimination and racism that suggested the three forms of microaggression and included perceptions of neighborhood disorder and racial discrimination, resiliency, stress, and social support, redlining, inadequate housing, poverty, violence, exposure to toxins and pollutants, and lack of social services and weight related discrimination during pregnancy. (12 p133) |
|  | **Bekkar, 2020 [1]**  A total of 57 studies (48 of 58 [84%] on air pollutants; 9 of 10 [90%] on heat) showed a significant association of air pollutant and heat exposure with birth outcomes. Positive associations were found across all US geographic regions. Exposure to PM2.5 or ozone was associated with increased risk of preterm birth in 19 of 24 studies (79%) and low birth weight in 25 of 29 studies (86%). (7 p1) |
|  | |
| **SOCIOCULTURAL ENVIRONMENT** | |
| Discrimination, racism, and stigma | **Amjad, 2019 [2]**  Previous research suggests that race‐based differences in adverse pregnancy  outcomes are the result of systemic racism faced by African American mothers. Race as a social institution perpetuates social stratification and ostracization which can potentially result in systematic exclusion of populations of adolescent mothers, putting them at a higher risk of poor maternal and birth outcomes. Maternal race has been suggested to operate through social hierarchies and the resulting differential access to economic structures, legal systems, education, employment opportunities, health care services by African American mothers. (18 p95) |
|  | **East, 2019 [19]**  Babies can have a low birthweight if they are born early, before 37 weeks gestational age, or are born small because the mother was poorly nourished through the pregnancy. Poverty can lead to malnutrition, unhealthy living environments, increased risk of infection, and increased stress in daily life. The social stigma associated with being marginalized in society is also a cause of stress. Babies whose growth has been restricted during pregnancy are more likely to die around the time of birth or to suﬀer from health problems. (19 p2) |
| Psychosocial stressors | **Chersich, 2020 [11]**  Lastly, cash transfers and other interventions which build resilience could assist pregnant women to access adaptive interventions such as cool, clean water, or reduce their need to earn income in the final weeks of pregnancy. (8 p9) |
|  | **Boccia, 2023 [9]**  Early life socioeconomic stressors can affect life course cardiometabolic, respiratory and mental health outcomes through epigenetic mechanisms or by influencing both fetal and childhood development and adaptation, and the differential burden of risk factors and health outcomes during early life. (5 p2) |
| Social and economic adversity and inequality | **Boccia, 2023 [9]**  According to Komro et al. 2019, the 12% reduction in LBW produced by the EITC translated into 3,760 fewer LBW babies born from Black mothers and 8,364 fewer LBW babies born from White mothers per year across the United States. Hispanic and non-Hispanic mothers displayed relatively similar effects. For minimum wage salaries instead, if all United States in 2014 had increased their minimum wages by 1 dollar there would likely have been an estimated 2,790 fewer LBW births for the year.  (5 p13) |
|  | **Dzekem,** **2024 [3]**  In recent years, issues of racial disparities and air pollution exposure have received increasing attention in the USA and globally. Trends in air pollution and racial disparities suggest worse exposure in people of color (POC) when compared to non-Hispanic whites (NHW). …They concluded that for all years and pollutants, the racial/ethnic group with the highest national average exposure was a racial/ ethnic minority group (NHB), with a national mean air pollution exposure higher for all three racial/ethnic minorities than for NHW. The degree of these disparities varied by pollutant and state. These findings are consistent with other studies that have explored this topic. (9 p540) |

**References**

1. Bekkar B, Pacheco S, Basu R, DeNicola N. Association of Air Pollution and Heat Exposure With Preterm Birth, Low Birth Weight, and Stillbirth in the US: A Systematic Review. JAMA Netw Open. 2020;3(6):e208243. doi: 10.1001/jamanetworkopen.2020.8243.

2. Amjad S, MacDonald I, Chambers T, Osornio-Vargas A, Chandra S, Voaklander D, et al. Social determinants of health and adverse maternal and birth outcomes in adolescent pregnancies: A systematic review and meta-analysis. Paediatr Perinat Epidemiol. 2019;33(1):88-99. doi: 10.1111/ppe.12529.

3. Dzekem BS, Aschebrook-Kilfoy B, Olopade CO. Air Pollution and Racial Disparities in Pregnancy Outcomes in the United States: A Systematic Review. J Racial Ethn Health Disparities. 2024;11(1):535-44. doi: 10.1007/s40615-023-01539-z.

4. Bowden ER, Chang AB, McCallum GB. Interventions to improve enablers and/or overcome barriers to seeking care during pregnancy, birthing and postnatal period for women living with vulnerabilities in high-income countries: A systematic review and meta-analysis. Midwifery. 2023;121:103674. doi: 10.1016/j.midw.2023.103674.

5. Khan Z, Vowles Z, Fernandez Turienzo C, Barry Z, Brigante L, Downe S, et al. Targeted health and social care interventions for women and infants who are disproportionately impacted by health inequalities in high-income countries: a systematic review. Int J Equity Health. 2023;22(1):131. doi: 10.1186/s12939-023-01948-w.

6. Atherton KM, Poupore NS, Clemmens CS, Nietert PJ, Pecha PP. Sociodemographic Factors Affecting Loss to Follow-Up After Newborn Hearing Screening: A Systematic Review and Meta-Analysis. Otolaryngol Head Neck Surg. 2023;168(6):1289-300. doi: 10.1002/ohn.221.

7. Chang R, Li C, Qi H, Zhang Y, Zhang J. Birth and Health Outcomes of Children Migrating With Parents: A Systematic Review and Meta-Analysis. Frontiers in Pediatrics. 2022;10. doi: 10.3389/fped.2022.810150.

8. Simonovich SD, Pineros-Leano M, Ali A, Awosika O, Herman A, Withington MHC, et al. A systematic review examining the relationship between food insecurity and early childhood physiological health outcomes. Transl Behav Med. 2020;10(5):1086-97. doi: 10.1093/tbm/ibaa021.

9. Boccia D, Maritano S, Pizzi C, Richiardi MG, Lioret S, Richiardi L. The impact of income-support interventions on life course risk factors and health outcomes during childhood: a systematic review in high income countries. BMC Public Health. 2023;23(1):744. doi: 10.1186/s12889-023-15595-x.

10. van Daalen KR, Kaiser J, Kebede S, Cipriano G, Maimouni H, Olumese E, et al. Racial discrimination and adverse pregnancy outcomes: a systematic review and meta-analysis. BMJ Glob Health. 2022;7(8). doi: 10.1136/bmjgh-2022-009227.

11. Chersich MF, Pham MD, Areal A, Haghighi MM, Manyuchi A, Swift CP, et al. Associations between high temperatures in pregnancy and risk of preterm birth, low birth weight, and stillbirths: systematic review and meta-analysis. Bmj. 2020;371:m3811. doi: 10.1136/bmj.m3811.

12. DiTosto JD, Holder K, Soyemi E, Beestrum M, Yee LM. Housing instability and adverse perinatal outcomes: a systematic review. Am J Obstet Gynecol MFM. 2021;3(6):100477. doi: 10.1016/j.ajogmf.2021.100477.

13. Crawford AD, Darilek U, McGlothen-Bell K, Gill SL, Lopez E, Cleveland L. Scoping Review of Microaggression as an Experience of Racism and Perinatal Health Outcomes. J Obstet Gynecol Neonatal Nurs. 2022;51(2):126-40. doi: 10.1016/j.jogn.2021.12.007.

14. Karger S, Bull C, Enticott J, Callander EJ. Options for improving low birthweight and prematurity birth outcomes of indigenous and culturally and linguistically diverse infants: a systematic review of the literature using the social-ecological model. BMC Pregnancy Childbirth. 2022;22(1):3. Epub 20220103. doi: 10.1186/s12884-021-04307-1.

15. Bellerose M, Collin L, Daw JR. The ACA Medicaid Expansion And Perinatal Insurance, Health Care Use, And Health Outcomes: A Systematic Review. Health Aff (Millwood). 2022;41(1):60-8. doi: 10.1377/hlthaff.2021.01150.

16. Behboudi-Gandevani S, Bidhendi-Yarandi R, Panahi MH, Mardani A, Paal P, Prinds C, et al. Adverse Pregnancy Outcomes and International Immigration Status: A Systematic Review and Meta-analysis. Ann Glob Health. 2022;88(1):44. doi: 10.5334/aogh.3591.

17. Cai C, Vandermeer B, Khurana R, Nerenberg K, Featherstone R, Sebastianski M, et al. The impact of occupational activities during pregnancy on pregnancy outcomes: a systematic review and metaanalysis. Am J Obstet Gynecol. 2020;222(3):224-38. doi: 10.1016/j.ajog.2019.08.059.

18. Heo S, Fong KC, Bell ML. Risk of particulate matter on birth outcomes in relation to maternal socio-economic factors: a systematic review. Environ Res Lett. 2019;14(12). doi: 10.1088/1748-9326/ab4cd0.

19. East CE, Biro MA, Fredericks S, Lau R. Support during pregnancy for women at increased risk of low birthweight babies. Cochrane Database Syst Rev. 2019;4(4):Cd000198. doi: 10.1002/14651858.CD000198.pub3.
